# Single quantum dot tracking reveals the impact of nanoparticle surface on intracellular state

**DOI:** 10.1038/s41467-018-04185-w

**Published:** 2018-05-08

**Authors:** Mohammad U. Zahid, Liang Ma, Sung Jun Lim, Andrew M. Smith

**Affiliations:** 1 Department of Bioengineering, University of Illinois at Urbana-Champaign, Urbana, IL 61801 USA; 2 Micro and Nanotechnology Laboratory, University of Illinois at Urbana-Champaign, Urbana, IL 61801 USA; 3 Department of Materials Science and Engineering, University of Illinois at Urbana-Champaign, Urbana, IL 61801 USA; 4 Present Address: Intelligent Devices and Systems Research Group, DGIST, 333 Techno Jungang-Daero, Hyeonpung, Daegu, 42988 Republic of Korea

## Abstract

Inefficient delivery of macromolecules and nanoparticles to intracellular targets is a major bottleneck in drug delivery, genetic engineering, and molecular imaging. Here we apply live-cell single-quantum-dot imaging and tracking to analyze and classify nanoparticle states after intracellular delivery. By merging trajectory diffusion parameters with brightness measurements, multidimensional analysis reveals distinct and heterogeneous populations that are indistinguishable using single parameters alone. We derive new quantitative metrics of particle loading, cluster distribution, and vesicular release in single cells, and evaluate intracellular nanoparticles with diverse surfaces following osmotic delivery. Surface properties have a major impact on cell uptake, but little impact on the absolute cytoplasmic numbers. A key outcome is that stable zwitterionic surfaces yield uniform cytosolic behavior, ideal for imaging agents. We anticipate that this combination of quantum dots and single-particle tracking can be widely applied to design and optimize next-generation imaging probes, nanoparticle therapeutics, and biologics.

## Abstract (web summary)

Quantum dots (QDs) mimic delivery agents for drugs and analytic compounds, but which route do they take inside cells? Here, the authors developed a technique to follow QDs, and they show that zwitterionic nanoparticle surface coatings make for the best delivery vehicle.

## Introduction

Pharmaceutical therapeutics and imaging agents composed of macromolecules and nanoparticles frequently require access to intracellular molecular targets, but delivery processes are inefficient and poorly understood^1–5^. Unlike small hydrophobic compounds, macromolecules are too large to passively transport through plasma membranes, and internalization leads to compartmentalization in endosomal vesicles that block access to cytoplasmic and nuclear machinery^1,6^. Chemical carriers that enhance cell uptake including peptides, polymers, and lipids result in the vast majority of payload trapped and clustered in vesicles^7,8^. Microinjection is highly effective, but too low in throughput for wide adoption, and membrane permeabilization techniques such as electroporation can substantially alter cell physiology^7^. Improved methods with high precision and throughput are urgently needed, and recent advances are promising^7,9,10^.

New mechanistic insights are needed to optimize the efficiency of intracellular delivery^4,7,11,12^, as downstream therapeutic outcomes are typically the only evaluation metric^13^. Snapshots of intracellular locations can be inferred from cell fractionation and fixed-cell imaging^13^, but because artifacts such as subcellular translocation can be overwhelming, live-cell techniques are strongly preferred^14,15^. Key insights have been derived from ensemble measurements in live cells using fluorescence correlation spectroscopy (FCS)^16^, fluorescence recovery after photobleaching (FRAP)^17^, and gross interpretation of diffuse haze patterns of cytosolic localization, compared with punctate vesicular localization^13,18^. Yet there remains no established method to quantitatively assay the states and distribution of intracellular cargo that is cytoplasmic or vesicular, and homogeneous or aggregated. However, single-fluorophore techniques are currently transforming our understanding of stochastic and heterogeneous molecular processes underlying cellular behaviors^19–22^, and provide a unique opportunity to assay the discrete events underlying intracellular delivery. Advances have been significantly benefitted by fluorescent quantum dots (QDs) as ultra-bright, photostable probes^23–28^, which simultaneously provide a platform to dynamically tune physicochemical properties that simulate broad classes of nanomaterials and biologics applied to intracellular targets.

Here we apply live-cell single-nanoparticle fluorescence imaging and tracking to quantitatively evaluate nanoparticle state distributions following intracellular delivery. We analyze intracellular QD trajectories to derive new classification metrics that distinguish distinct intracellular states that have previously been inaccessible through ensemble methods with the goal of mechanistically evaluating intracellular delivery of nanoparticles and macromolecules. We use multidimensional analysis of diffusion rate, confinement, and brightness to quantify nanoparticle uptake, cluster distribution, and cytosolic numbers in single cells. We show that small subpopulations can be measured amid a haze of primarily vesicular nanoparticles. QDs with differing physicochemical surfaces lead to vastly different distributions, although absolute counts of the cytoplasmic nanoparticles are largely independent of surface. We show that achieving single, cytoplasmic QDs requires colloidal stability through strong binding polymers and nearly neutral electrostatic charge. QDs with zwitterionic surfaces are the most mobile and homogeneously dispersed after delivery, adding to the rapidly expanding utility of zwitterionic nanomaterials.

## Results

### Quantum dot surface properties

**Fig. 1:**
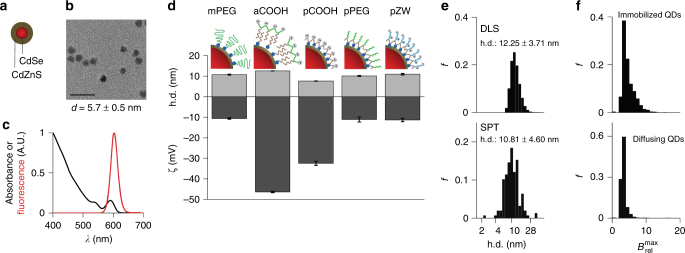
Characterization of quantum dots with different coatings. **a** Schematic depiction of (core)shell (CdSe)CdZnS quantam dots (QDs). **b** Transmission electron micrograph of QDs; scale bar = 20 nm. **c** Absorbance and fluorescence spectra of QDs in hexane. **d** Schematic depictions of each QD coating (not to scale), showing differences in the number of the binding groups (blue circles) per ligand. The coating-naming convention indicates the binding mode (m = monodentate, a = amphiphilic, p = polydentate). Hydrodynamic diameter (h.d.) measured by dynamic light scattering (DLS) and zeta potential (ζ) were acquired in pH 7.4 buffer. *n* = 3 for all QD coatings, and all error bars indicate s.e.m. **e** Comparison of h.d. for mPEG-QDs either by DLS in aqueous solution or single-particle tracking (SPT) in 98% glycerol. **f** Distributions of maximum single-particle relative brightness ($B_{{\mathrm{rel}}}^{{\mathrm{max}}}$) for QDs immobilized on a glass coverslip or diffusing in 98% glycerol, demonstrating uniform brightness in a cell-free environment. *f* = frequency

We synthesized QDs with diverse surfaces based on quasi-spherical (core)shell (CdSe)CdZnS nanocrystals (Fig. 1a), with 5.7 nm diameter (Fig. 1b) and 605 nm fluorescence emission (Fig. 1c). QDs were coated with five different polymeric coatings, depicted schematically in Fig. 1d (detailed structures are in Supplementary Fig. 1), with hydrodynamic diameter (h.d.) and electrostatic charge (as zeta potential, ζ). The coatings were prepared to satisfy particular design criteria spanning a broad range of electrostatic charges and stabilities to allow specific pairwise comparisons. Importantly, all were compact, with h.d. between 7 and 12 nm, to simulate protein therapeutics and to avoid the expected cytoplasmic sieving threshold of ~20–30 nm^17,29^. Coatings were based on monodentate thiol-terminated polyethylene glycol (mPEG), a series of polydentate ligands with tunable hydrophilic groups including carboxylic acids (pCOOH), polyethylene glycol (pPEG), and zwitterions (pZW), and amphiphilic polymers functionalized with carboxylic acids (aCOOH). While the h.d. values were only slightly different, the electrostatic charges were substantially different, with ζ between –30 and –45 mV for COOH-functional coatings due to deprotonation at physiological pH, and near to –10 mV for coatings based on PEG and zwitterions. All QDs were colloidally stable for months^30,31^, with the exception of mPEG-QDs, as monodentate ligands slowly detach upon dilution in oxidizing conditions, although these QDs were stable in concentrated stock solutions for several days (Supplementary Fig. 2). Diffusive behavior based on ensemble measurements using dynamic light scattering (DLS) or liquid chromatography was similar to that measured by single-particle tracking (SPT) in viscous solution (Fig. 1e). In addition, the per particle fluorescence brightness for isolated QDs was similar when immobilized or diffusing in solution (Fig. 1f), however surface deposition led to brighter populations, indicative of clusters.

### Intracellular delivery

**Fig. 2:**
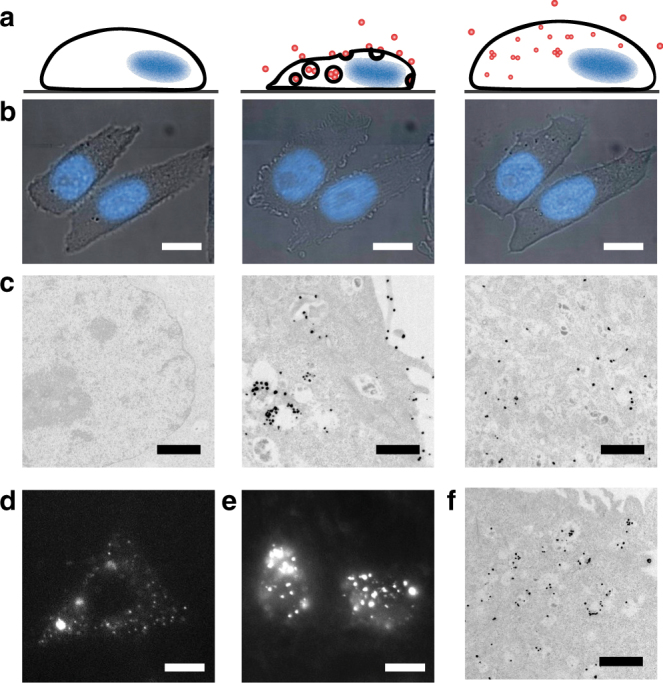
Quantum dot delivery to CHO cells by osmotic pinosome lysis. **a** Schematic of osmotic pinosome lysis (OPL), depicting changes in cell morphology and location of quantum dots (QDs) for cells prior to treatment, during hypertonic loading of QDs, and after hypotonic treatment to rupture pinosomes. **b** Brightfield images with nuclear stain (blue) demonstrating morphological changes of cells undergoing OPL, at stages corresponding to schematics in **a**. **c** Transmission electron microscopy (TEM) images of OPL-mediated delivery of QDs, corresponding to schematics in **a**, where the particles can be seen primarily trapped in the endosomes, and then dispersed in the cytoplasm. **d** Fluorescence HILO image of QDs delivered via OPL and **e** via passive uptake. **f** TEM image of cells after passive uptake. Scale bars are 10 μm for light microscopy images and 1 μm for TEM images. Additional TEM images are in Supplementary Fig. 3

QDs were delivered to cultured cells using osmotic pinosome lysis (OPL)^32^, in which fluid-phase pinocytosis facilitates vesicle-mediated cell entry and pressure-driven cytoplasmic release, yielding efficient cytoplasmic delivery of proteins. In this two-step process, depicted in Fig. 2a, the cells were incubated with a hypertonic aqueous buffered solution for 10 minutes. This solution contained sucrose, PEG, and QDs, inducing fluid-phase pinocytosis and cell volume loss due to osmotic water efflux. QDs in intracellular pinosomes are then released due to pressure-induced vesicular rupture upon 3 min exposure to a hypotonic solution. Cells were imaged within 45 min. We verified cell volume changes by brightfield microscopy (Fig. 2b) and verified QD internalization by transmission electron microscopy (TEM) after silver development (Fig. 2c and Supplementary Fig. 3). By fluorescence microscopy (Fig. 2d), the diffuse intracellular pattern of primarily single QDs was distinct from cells in which QDs were simply added in the cell culture medium (Fig. 2e), which resulted in bright, immobile punctate spots, revealed to be endosomal QDs by TEM (Fig. 2f).

### Single-particle imaging and analysis

**Fig. 3:**
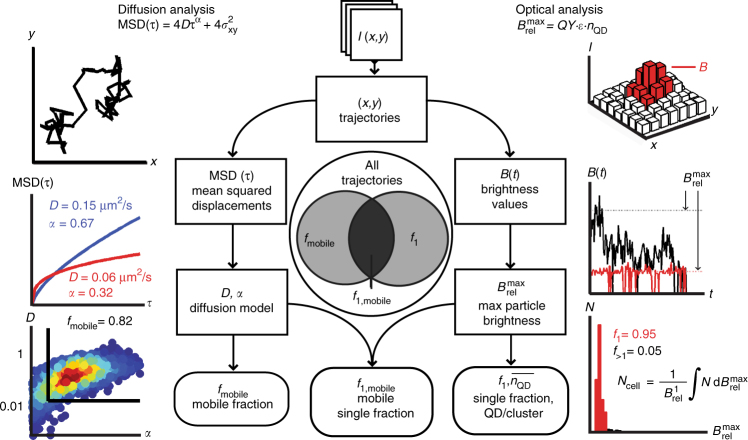
Analysis methodology for quantam dot mobility and clustering. Single particle trajectories (*x*(*t*),*y*(*t*)) are extracted from raw image stacks of *I*(*x*,*y*), and each trajectory is analyzed by diffusion and the optical metrics. For diffusion analysis, mean squared displacement (MSD) curves for each trajectory are fit to an anomalous diffusion model to yield a diffusion coefficient, *D*, and confinement parameter, *α*. These values for each trajectory are aggregated across all cells for a given experimental condition, and thresholds are imposed to determine the mobile fraction, *f*_mobile_. Representative data are shown at left and a heat map of *D* versus *α* shows an example of a high mobile fraction (*f*_mobile_ = 0.82). For optical analysis, the brightness per trajectory at each time point, *B*(*t*), is analyzed to determine its maximum relative value, $B_{{\mathrm{rel}}}^{{\mathrm{max}}}$, to calculate the number of QDs per cluster, *n*_QD_. Representative data show intensity time traces of a single quantum dot (red) and a cluster (black). The distribution of all $B_{{\mathrm{rel}}}^{{\mathrm{max}}}$ values for each cell is then analyzed to extract the fraction that is single, *f*_1_, and the average number of quantum dots per cluster, $\overline {n_{{\mathrm{QD}}}}$, is then aggregated across all cells for each experimental condition. Representative data show a $B_{{\mathrm{rel}}}^{{\mathrm{max}}}$ distribution with a high single fraction (*f*_1_ = 0.95) and low cluster fraction (*f*_>1_ = 0.05). The diffusion and optical analyses are then combined to analyze specific particle populations to determine the fraction that are both single and mobile, *f*_1,mobile_, represented in the Venn diagram at the center. Parameters in rectangles are determined for each trajectory, whereas the parameters in rounded boxes are determined for a QD population in one cell or multiple cells

Intracellular QDs were imaged using highly inclined laminar optical sheet (HILO) microscopy^33^, in the presence of complete medium containing cell-impermeable bromocresol green (BCG) that quenched extracellular QDs (Supplementary Fig. 4)^34^. Single-particle videos were analyzed by SPT using MATLAB u-track software^35^, and each particle trajectory was separately analyzed for diffusion and optical brightness (Fig. 3). For diffusion analysis, a mean squared displacement (MSD) curve by time increment *τ* was fit to a model of anomalous diffusion^36,37^, yielding a diffusion coefficient *D* and unitless confinement parameter *α*, according to the equation

1 $$\rm{MSD}\left( \tau \right) = 4D\tau ^\alpha + 4\sigma _{xy}^2$$

where *σ*_xy_ is the average localization error of the trajectory. For    sub-Brownian (subdiffusive) motion, *α* is less than 1, reflecting conventional cytosolic thermal motion that is consistent with our analysis (see below). State threshold values for mobility (*D* > 0.020 μm^2^/s and *α* > 0.21), established empirically, were applied to calculate the fraction of particles that were mobile in a cell, *f*_mobile_. For optical analysis, we measured brightness as a function of time for each trajectory to calculate the number of QDs per cluster, *n*_QD_, by

2 $$B_{{\mathrm{rel}}}^{{\mathrm{max}}} = QY \cdot \varepsilon \cdot n_{{\mathrm{QD}}} = B_{{\mathrm{rel}}}^1 \cdot n_{{\mathrm{QD}}}$$

where *QY* is the quantum yield, *ε* is the extinction coefficient, and $B_{{\mathrm{rel}}}^1$ is the relative single-QD brightness in the on-state, which is homogeneous for uniform QDs with this composition^38^. Because QDs randomly fluctuate between on and off states (blinking), only the brightest state was used to calculate $B_{{\mathrm{rel}}}^{{\mathrm{max}}}$. $B_{{\mathrm{rel}}}^1$ was extracted from single-cell $B_{{\mathrm{rel}}}^{{\mathrm{max}}}$ distributions for each cell to calculate the total number of QDs per cell, *N*_cell_, the fraction of single, unaggregated QDs, *f*_1_, and other derived metrics. The two analyses were combined to calculate the fraction of QDs that are both single and mobile, *f*_1,mobile_, which is the desired state for most applications.

### Impact of physicochemical properties

**Fig. 4:**
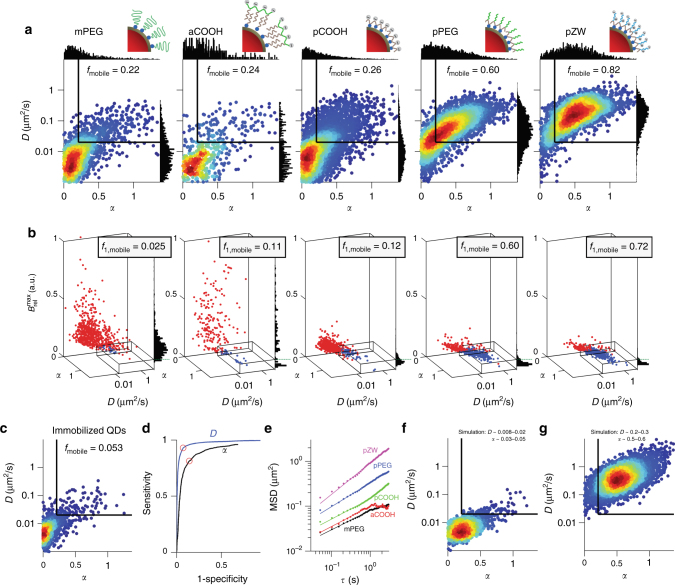
Coating-dependent behavior of intracellular quantum dots. **a** Heat maps of diffusion coefficient, *D*, versus confinement parameter, *α*. Histograms of *D* and *α* are projected on the *x* and *y* axes, respectively, and thresholds imposed for mobility are shown as black lines, yielding *f*_mobile_ shown in each plot. *n* = 7, 7, 16, 11, and 18 CHO cells for mPEG, aCOOH, pCOOH, pPEG, and pZW, respectively. **b** Representative 3D plots of *D* versus *α* versus $B_{{\mathrm{rel}}}^{{\mathrm{max}}}$ for the different quantum dot (QD) coatings in individual cells. The box indicates the same threshold from **a**, in addition to a $B_{{\mathrm{rel}}}^{{\mathrm{max}}}$ threshold for delineating populations of single QDs to determine *f*_1,mobile_, shown in the inset of each plot. **c** Heat map of *D* versus *α* for immobilized QDs. **d** Receiver operating characteristic (ROC) curves distinguishing immobilized QDs from pZW-coated QDs in CHO cells, independently for *D* (blue) and *α* (black). Cutoffs that maximize both sensitivity and specificity (*D* > 0.020 μm^2^ s^−1^ and *α* > 0.21) are marked by red circles. **e** Ensemble average MSD plot for all recorded trajectories fit to Eq. (1) for the first 20 time increments. Calculated *D* and *α* values from ensemble and single-trajectory analyses are in Supplementary Table 1. **f** Heat map of *D* versus *α* for simulated trajectories generated using *D* and *α* inputs across the indicated ranges to approximate the mPEG-QD distribution. **g** Heat map of *D* versus *α* for simulated trajectories generated using *D* and *α* inputs across the indicated ranges to approximate the pZW distribution

Figure 4a shows the distribution of trajectory diffusion parameters from SPT data for QDs with different coatings delivered to Chinese hamster ovarian cancer (CHO) cells, using OPL (representative videos are provided as Supplementary Movies 1–5). Heat maps of *D* versus *α* demonstrate the impact of surface coating on intracellular mobility and the freedom to explore regions of a cell larger than a pinosome (460 ± 150 nm, mean ± s.d., diameter by TEM measurements). Two distinct mobility states of QDs are clearly distinguished. Based on empirical thresholds, the majority of detected particles were immobile for mPEG, which was electrostatically neutral and unstable, as well as for aCOOH and pCOOH coatings, which were both stable but strongly charged. QDs coated with pPEG and pZW demonstrated a strikingly different mobility trend, with 60–82% of trajectories within the mobile range, demonstrating that stability and neutrality are both required for mobility. Note that these distinct states are not apparent from single-dimensional analysis of either *D* or *α* alone, shown as histogram projections on the graph axes.

Figure 4b shows 3D scatter plots of $B_{{\mathrm{rel}}}^{{\mathrm{max}}}$ for each particle type together with both mobility parameters. Each QD coating class exhibited a distinct level of clustering, indicated by the distributions of points along the *z-*axis. The rectangular box in the bottom right of each plot indicates the region designated to those that are both mobile and single (*f*_1,mobile_), with trajectories color-coded to indicate whether they fall in the brightness population of single QDs (blue) or clusters (red). The mPEG and aCOOH QDs demonstrated substantial clustering inside the cells, indicated by numerous red spots at high $B_{{\mathrm{rel}}}^{{\mathrm{max}}}$ values. However, pPEG, pZW, and the mostly immobile pCOOH QDs all had *f*_1_ fractions greater than 45%. These are single-cell data; Supplementary Fig. 5 shows representative cell-to-cell variability.

The mobility thresholds were derived from comparisons between immobilized QDs on glass surfaces (Fig. 4c) and intracellular pZW-QDs, and receiver operating characteristic curves are shown in Fig. 4d. The span of distinguishable mobile fraction ranged from 5.3 to 82%. Figure 4e shows the pooled *MSD*(τ) curves for all trajectories within each coating class, showing the clear distinguishable differences between the different materials at the ensemble level. Mean *D* values spanned between 0.02 and 0.2 μm^2^ s^−1^ and were similar when calculated from individual trajectories or as an ensemble, however the average *α* was substantially smaller when calculated per trajectory (0.26–0.49), compared with the pooled data (0.44–0.70) (Supplementary Table 1). The values calculated from individual tracks are presumably more accurate, as each trajectory was fit using a measured localization error for each track, which could span a broad range of values between tracks. We corroborated these findings with FCS on HeLa cells loaded with pPEG-QDs (Supplementary Fig. 6 and Supplementary Table 2). The average *D* measured with SPT was 0.10 μm^2^/s with *α* = 0.45, while the ensemble average for FCS was *D*=0.84 μm^2^/s and *α* = 0.66. FCS also showed a wide range of *D* values in different cell regions, as well as multimodal behavior in single locations. However, highly immobile populations were not clearly resolved with this technique, which is likely the source of the small discrepancy between the two average *D* values, together with differences in time lag at which each is defined (1 s for SPT and τ_D_ for FCS).

As shown in Fig. 4e–f, we further simulated anomalous diffusion trajectories for QD classes at the extremes of mobility, and analyzed the impact of track length on *D* versus *α* heat maps (Supplementary Figs. 7–10). From these analyses, it is clear that the 45° tilt to the heat map distribution is not due to a heterogeneous population, but rather is an intrinsic pattern derived from short track lengths. Also when fitting the experimental data to simulations, it is clear that the distributions observed in cells are too broad to reflect a single population, instead requiring a range of mobility parameters. An analysis of the error associated with *D* versus *α* values, derived from the simulated data, shows that both values are fairly accurate, except when *D* is small such that the mobility is in the same range as the localization error (Supplementary Fig. 11).

### Metrics for intracellular delivery analysis

**Fig. 5:**
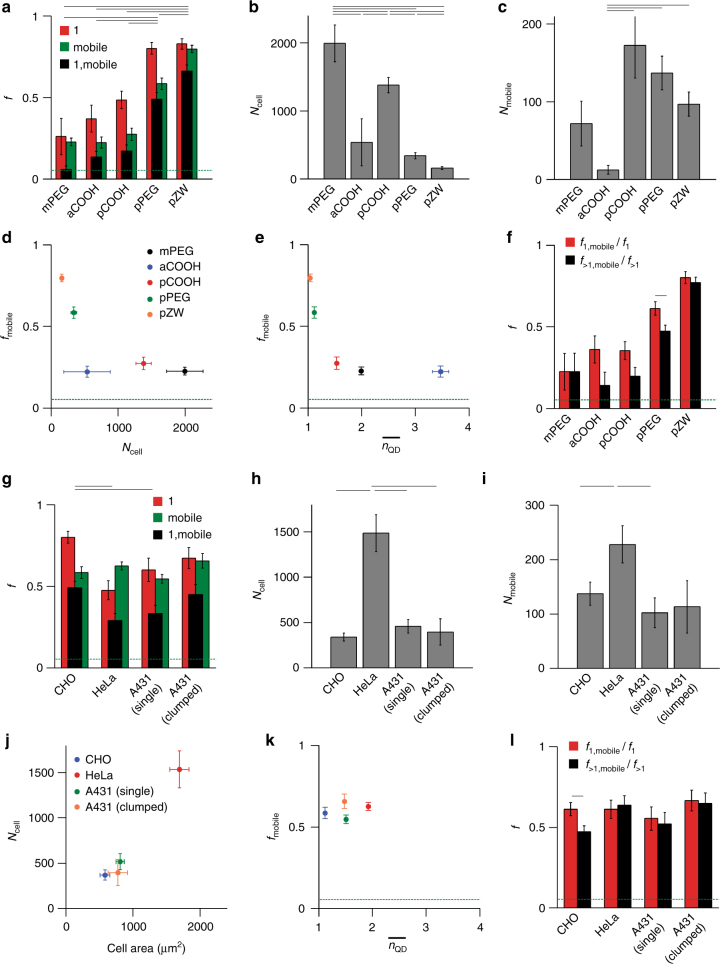
Single-cell metrics comparing QD coatings and cell types. **a** Data for each QD coating show *f*_1_, *f*_mobile_, and *f*_1,mobile_. The green dashed line in **a**, **d**–**g**, **k**, **l** indicates the lower limit of *f*_mobile_ (0.053). Horizontal black lines indicate *p* < 0.05, calculated using Student’s *t* test, for *f*_1,mobile_ comparisons. **b** Number of internalized QDs per cell for 40 nM loading concentrations. For mPEG and pCOOH, data are linear extrapolations from 10 nM concentrations due to high spot intensity due to clustering. **c** Mobile QDs per cell. **d** Negative correlation between *f*_mobile_ and *N*_cell_ for coatings demonstrates that materials with high mobile fractions deliver less efficiently. **e** Negative correlation between *f*_mobile_ and $\overline {n_{{\mathrm{QD}}}}$demonstrates that QDs are clustered in cells in which they are immobile. Coatings are indicated by color codes in **d**. Additional *n*_QD_ distribution weightings are in Supplementary Figs. 12 and 13. **f** Comparison of *f*_1,mobile_/*f*_1_ and *f*_>1,mobile_/*f*_>1_ reflect the mobility for single QDs and clusters, respectively. Horizontal lines indicate *p* < 0.05 within coating types only. For QD coating comparisons, *n* = 7, 7, 16, 11, and 18 cells for mPEG, aCOOH, pCOOH, pPEG, and pZW, respectively. **g** Data for three cell types (A431, CHO-K1, and HeLa), showing *f*_1_, *f*_mobile_, and *f*_1,mobile_. For A431 cells, single and clumped cells were analyzed separately. Horizontal lines indicate *p* < 0.05 for *f*_1,mobile_. **h** Number of internalized QDs per cell. **i** Mobile QDs per cell. **j** *N*_cell_ correlates with the average cell area. **k** Little correlation is observed between *f*_mobile_ and $\overline {n_{{\mathrm{QD}}}}$, but different cell types have distinct degrees of clustering. Cell types are indicated by color codes in **j**. **l** Comparison of *f*_1,mobile_/*f*_1_ and *f*_>1,mobile_/*f*_>1_ reflect the mobility for single QDs and clusters, respectively. Horizontal lines indicate *p* < 0.05 within cell types only. All cell-type comparisons used pPEG-QDs, with *n* = 10, 9, 7, and 9 cells for CHO, HeLa, single A431, and clumped A431 cells, respectively. All error bars are s.e.m. Comprehensive *p* values are in Supplementary Tables 3–12

Aggregated trends for *f*_1_, *f*_mobile_, and *f*_1,mobile_ are summarized for each QD coating in Fig. 5a. The pPEG and pZW coatings yielded the highest degree of cytoplasmic freedom as well as the least amount of aggregation, while the mPEG, aCOOH, and pCOOH coatings led to both clustering and low mobility. SPT provides numerous analytical metrics that can be used to determine the origin of these effects, as described below.

Uptake efficiency: We calculated the total number of QDs per cell, *N*_cell_, by using the relative single-QD brightness, $B_{{\mathrm{rel}}}^1$, to convert the $B_{{\mathrm{rel}}}^{{\mathrm{max}}}$ value for each trajectory to QD number, shown in Fig. 5b. The theoretical value of *N*_cell_ should range from 100 to 150 for all QDs, independent of coating, based on the purported delivery mechanism in which extracellular fluid is simply transported into the cell with an influx volume of ~10 fL in the hypertonic loading step^32^. The *N*_cell_ for pZW-QDs (159 ± 20) was indeed close to the expected value, suggesting that pure fluid transport was responsible for uptake, but higher *N*_cell_ values for all other QDs, reaching 1963 ± 270 for mPEG-QDs, indicated a parallel secondary uptake process, likely via membrane adsorption. This was corroborated by TEM imaging studies that showed membrane-bound mPEG-QDs during loading (Supplementary Fig. 3b) and an excess of QDs per vesicle (6.5 ± 1.3), compared to expectations (1.2) for fluid-phase pinocytosis of 40 nM extracellular QDs. Furthermore, we repeated the delivery of mPEG-QDs in the presence of adsorption-blocking agents (casein) and observed a vastly different diffusion pattern (Supplementary Movies 6 and 7). *N*_cell_ decreased to 23% of its value without blocking agents (*p* = 0.036), and *f*_mobile_ increased by 77% (*p* = 0.018 by  Student’s *t* test) (Supplementary Fig. 14).

Endosome release efficiency: We calculated the number of completely mobile cytoplasmic QDs per cell, *N*_mobile_. The theoretical value of *N*_mobile_ should be the same as *N*_cell_ if OPL efficiently ruptures endosomes. Figure 5c shows a surprising result that despite the uptake varying by more than 12-fold, *N*_mobile_ was statistically indistinguishable for QDs coated with mPEG, pCOOH, pPEG, and pZW, with *N*_mobile_ spanning 72–172, similar to the expected value for the pure fluid-phase transport. Importantly, we were able to distinguish these mobile populations amid a background of immobile particles that were sometimes 20 times more abundant. For mPEG-QDs, *N*_mobile_ did not change significantly with the addition of blocking agents during delivery (*p* = 0.60 by Student's *t* test; Supplementary Fig. 14) despite a drastic reduction in *N*_cell_, presumably because the osmotic transport that yields mobile cytoplasmic QDs is not mediated by adsorption. Using these population distributions, we observed that *f*_mobile_ was negatively correlated with *N*_cell_, as shown in Fig. 5d, demonstrating that endosomal release is inefficient in cells with large *N*_cell_. For pZW, *f*_mobile_ was 82% and was nearly the same as diffusing QDs in liquid (Supplementary Fig. 15), suggesting that pZW-QDs were fully released in the cytoplasm. Note that *N*_mobile_ for aCOOH-QDs was significantly smaller than that of all neutral QDs (*p* < 0.05), likely due to the strong anionic charge that can electrostatically repel the plasma membrane during loading.

Correlation between mobility and clustering: To gain insights into how internalization differs between the coatings, we separately analyzed single QDs and clusters to determine how clustering impacts mobility. Figure 5e shows the average cluster sizes, $\overline {n_{{\mathrm{QD}}}}$, (alternative distribution weightings in Supplementary Figs. 12 and 13), showing that clustering is associated with lower mobility. Figure 5f shows plots of *f*_1,mobile_/*f*_1_ and *f*_>1,mobile_/*f*_>1_. For coatings for which *f*_1,mobile_/*f*_1_ is greater than  *f*_>1,mobile_/*f*_>1_, we can infer that clustering restricts mobility, either because clusters are too large to exit the endosomes, or because the endosomes cannot rupture. This was indeed the case that clustering restricted mobility for pCOOH, aCOOH, and pPEG QDs (*p* < 0.07 by Student’s *t* test), all of which loaded well beyond the limits of osmotic transport, likely due to adsorption to membrane components. However, for both pZW and mPEG, *f*_1,mobile_/*f*_1_ and *f*_>1,mobile_/*f*_>1_ were statistically indistinguishable (*p* > 0.5). This result is logical for pZW-QDs, which were nearly entirely mobile, but this suggests a unique uptake pathway for mPEG QDs that prevented endosomal release, even when internalized individually in single endosomes. These QDs were unstable at low concentrations, so adsorption to endosomes likely prevented endosomal escape.

Cell type comparisons: We analyzed the impact of cell type using CHO cells, HeLa human cervical cancer cells, and A431 human epidermoid cancer cells, the latter of which were analyzed separately for single cells and cells that grew in clumps with cell–cell junctions that reduce the accessible membrane surface area. We tested pPEG-QDs, which showed a mix of both cytoplasmic and immobile states in our initial coating analysis in CHO cells. As shown in Fig. 5g, cell type had less impact on *f*_1_, *f*_mobile_, and *f*_1,mobile_ compared with the impact of QD coating, which is consistent with previous ensemble studies of OPL^32^. However, *f*_1_ was significantly larger for CHO cells, which may reflect the distinct pinocytotic mechanisms due to their small size and surface area^39^, whereas HeLa cells exhibited significantly lower *f*_1_, higher *N*_cell_ (Fig. 5h), and higher *N*_mobile_ (Fig. 5i), consistent with higher adsorption-dependent uptake and their larger surface area (Fig. 5j). Cell type had a significant impact on clustering that likewise correlated with the membrane surface area (Fig. 5k). Only the smallest CHO cells exhibited enhanced endosomal escape for single QDs compared to clusters (*f*_1,mobile_/*f*_1_ > *f*_>1,mobile_/*f*_>1_), shown in Fig. 5l.

## Discussion

These new classes of single-particle analysis metrics provide unique insights into the physical state of intracellular nanoparticles, as well as the mechanisms by which they enter cells and access the cytoplasm. Two metrics in particular, *N*_cell_ and *f*_mobile_, reflect the efficiency of transport into the cell and the efficiency of endosomal release, respectively, the two critical steps in cytoplasmic delivery, whereas derived metrics *n*_QD_, *f*_1_, and *f*_1,mobile_ yield important insights into the state of the internalized materials and the endosomal release mechanism. These outcomes require distinctive photophysical properties of QDs, with bright and stable emission that is homogeneous across a population of nanoparticles^38^. These features allow registration of brightness to particle stoichiometry as well as long-term tracking, which are not possible with organic dyes^40^.

QDs also provide the ability to widely tune colloidal physicochemical properties due to the diverse range of surface coatings that have been developed^41,42^. We used thin coatings to yield similar h.d. to therapeutic proteins like antibodies, polysaccharides, and micelles with typical zeta potentials near –10 mV. It is clear from our analyses that pZW-QDs are uniquely suited to cytoplasmic delivery through OPL. The pPEG-QDs had similar physical properties, but smaller *f*_mobile_, likely due to nonspecificbinding evident from higher *N*_cell_. The differences in mobility between the pZW and pPEG QDs, and the increased efficiency of endosomal escape for the pZW-QDs may be due to the different levels of hydration and different interactions with intracellular proteins. Molecular simulations comparing zwitterionic and nonionic PEG-based materials have shown that zwitterionic materials exhibit stronger hydration than nonionic PEG materials, and that PEG materials interact with hydrophobic domains of proteins, while zwitterionic materials have limited interaction with these domains^43^. However, *f*_mobile_ for pPEG-QDs increased substantially when delivered through liposomal vesicles, reaching 78% (Supplementary Fig. 16), demonstrating that adsorption processes occurring during delivery can have a dominating impact on the intracellular state. When PEG was only weakly attached (mPEG-QDs), *N*_cell_ drastically increased for OPL delivery, while endosomal escape drastically reduced. Proteins that exhibit membrane adsorption were found to exhibit similar outcomes^44^. Uptake was further enhanced when the magnitude of electrostatic charge was greater (aCOOH-QDs and pCOOH-QDs), likely due to binding to cationic protein domains, but endosomal release was further diminished, likely due to rapid acidification of vesicles and neutralization of the ionically stabilized colloids^45,46^. This is consistent with the low efficacy of OPL-mediated gene delivery of anionic nucleic acids^32^ and their complexes with cationic polymers^47^, compared with the high delivery efficacy of proteins, which are zwitterionic colloids with physicochemical similarities to pZW-QDs. In fact, anionic nucleic acids are effectively immobile in cells with molecular weights >100 kDa, whereas neutral colloids of equal mass are mobile^29^. Notably, we did not investigate cationic materials due to their high adsorption strength to cellular structures^48^. These outcomes suggest that for intracellular targeting with molecular probes, stable and neutral surfaces are ideal.

Based on these results, it is evident that total cell uptake measurements are not suitable for optimizing cytoplasmic delivery because the absolute number of delivered cargo *N*_cell_ is highly disproportional to *N*_mobile_, as QDs with smallest *N*_cell_ values (pPEG-QDs and pZW-QDs) had similar *N*_mobile_ values to those with the largest *N*_cell_ values (mPEG-QDs). Importantly, these live-cell analyses were much more quantitative compared with the analysis of TEM images, which exhibited significant fixation artifacts. In particular, QDs were often observed near the cell periphery (Supplementary Fig. 3c), which was not reflected in living cells. Moreover, different QD chemical groups elicit substantially dissimilar interactions with fixatives and permeabilization solvents, so accurate materials comparison are not possible in fixed cells^14^.

New imaging advancements could further be applied to gain additional insights. For analyzing the total uptake, *N*_cell_ is estimated based on fluorescence signal from a focal plane that is thinner than the cell height. While this is sufficient for comparing between different nanomaterials in the same cell types, absolute values could be measured by simultaneous acquisition of multiple focal planes^49^, but with substantially increased analysis time. *N*_cell_ is also based on the net fluorescence signal from clusters, which may exhibit self-quenching if QDs are in close proximity. We do not believe this to be the case because the inter-QD Forster distance (~6.6 nm)^50^ is much smaller than the shortest distance possible for these QDs with >2 nm coatings (~10 nm), for which 11% quenching is the theoretical maximum. Indeed, atomistic calculations predict that electronic coupling is nearly zero at these distances^51^. Furthermore, brightness values are clearly quantized in cells with low autofluorescence (Supplementary Fig. 17). Measuring the excited state lifetime could provide additional insights as to the degree of electronic coupling in clusters.

It is important to evaluate experimental *D* and *α* distributions from SPT in the context of anomalous diffusion trajectory simulations. The spread of the measured values strongly depends on track length (Supplementary Fig. 7), so we used simulated tracks truncated to the lengths of the experimental track length distributions to distinguish the degree to which data spread derives from the fitting or from underlying physical dispersions (Supplementary Fig. 9, 10). The results showed that data spread was greater than that of a monodisperse population, requiring a range of *D* and *α* values to account for even the most homogeneous pZW-QD data. Average *D* and *α* values could also significantly deviate from the real values, depending on both track length and the fitting (Supplementary Fig. 11), especially when *D* and *α* were small, and when the spot localization error becomes significant. The slowest populations were also challenging to quantitatively characterize with FCS, which showed a similar range of *D* and *α* values as SPT (Supplementary Fig. 6), but very slow populations could not be observed. Doing so would require >10 minutes of data acquisition that would be highly phototoxic to cells. FCS in general can be challenging to interpret as polydispersity and anomalous diffusion cannot be independently distinguished in time-traces^52^, and single-QD intermittency can interfere with time-correlation analyses^53^. The ability of single-particle techniques to provide direct insight into particle heterogeneity is thus a major benefit, compared with ensemble techniques. The accuracy of measured *D* and *α* values can be improved by using a faster video frame rate or methods that extend the z-range to increase track lengths, and by using brighter QDs to increase the photon flux to reduce localization error. However brighter emitters would necessitate the use of larger sizes that may no longer physically reflect proteins being emulated.

With recent advances in instrumentation, probes, and image analysis software, the capacity to perform single-molecule tracking is now widely available^19–22,54^. QDs are an important component in this toolbox, and are routinely used for single-molecule tracking of membrane proteins and dynamic processes in cell environments^23–28,55^. Our analysis techniques and surface modulation methods can be widely adopted to improve our understanding of cell delivery processes^4,7,11^, which are plagued by the need to balance the seemingly opposing processes of endocytosis and endosomal escape, both of which can now be independently assessed in single cells. With these metrics, it may be further possible to accurately apply pharmacodynamic models relating dose to efficacy and potency for nanoparticle therapeutics, which present unique challenges due to the uncertainty of numerous transport parameters^56^. Furthermore, zwitterionic QDs possess a unique potential as probes for evaluating cytoplasmic processes, and could be used to analyze the behavior of specific intracellular molecules, if they can be precisely targeted without impairing the function of the molecule. Finally, this method is particularly well suited for imaging cultured cells, but can also be applied to evaluate delivery in living tissues in both intracellular and extracellular domains using imaging techniques with both rapid acquisition and high depth penetration such as spinning disk confocal microscopy, light sheet microscopy, or holographic multiphoton imaging^57,58^.

## Methods

### Quantum dot synthesis

Nanocrystals composed of (core)shell (CdSe)CdZnS were synthesized in organic solvents^38^. In sequential steps, 3.0 nm CdSe cores were synthesized and shells composed of 3.2 monolayers of CdS and 1.5 monolayers of ZnS were grown layer-by-layer. The resulting nanocrystals were coated with oleylamine and oleic acid, and were purified by acetone precipitation and hexane-methanol extractions. Coating with pPEG^30^, pCOOH^30^, pZW^59^, and aCOOH^38^ was performed according to our previously reported methodologies described in Supplementary Methods. For coating with mPEG, QDs in hexane were transferred to *N*-methylformamide using tetramethylammonium hydroxide^30^ and mixed with a 5000-fold molar excess of methoxy-PEG-SH (2000 Da; Rapp Polymere) at 60 °C under nitrogen atmosphere with stirring for 3 h. The QDs were then precipitated using anhydrous diethyl ether, dispersed in methanol, and precipitated again with a mixture of hexane and chloroform. The nanocrystals were then dispersed in 50 mM sodium borate buffer and centrifuged to remove possible aggregates. These QDs were stable for more than one week under ambient conditions at >1 μM concentration (Supplementary Fig. 2).

### Cell culture and quantum dot delivery

A431, CHO, and HeLa cells (ATCC) were seeded at a density of 20,000 cells/cm^2^ in LabTek chambers (Thermo Scientific), 24 h before OPL treatment. CHO cells were cultured in Kaighn’s Modification of Ham’s F-12 Medium (Cell Media Facility, School of Chemical Sciences, UIUC). A431 cells and HeLa cells were cultured in Dulbecco’s Modified Eagle’s Medium (DMEM; Cell Media Facility, School of Chemical Sciences, UIUC) with 10% fetal bovine serum (FBS; HyClone) and 1% penicillin/streptomycin (P/S; Mediatech). Cells were washed twice with phosphate buffered saline, and the hypertonic loading reagent (Life Technologies) containing QDs (10 or 40 nM) was added. Cells were incubated for 10 min at 37 °C, and then the medium was removed and replaced with hypotonic lysis medium composed of six parts incomplete DMEM without phenol red and four parts deionized water for 3 min at 37 °C. The medium was then replaced with complete DMEM without phenol red and the cells were incubated for 10 min at 37 °C. Nuclei were stained with Hoechst (Sigma-Aldrich) for 20 min, followed by washing and treatment with BCG (200 μM; Sigma-Aldrich) in phenol red-free DMEM to quench any extracellular QDs, and cells were imaged within 45 min. The protocol was optimized to ensure that the majority of cells were viable (Supplementary Fig. 18). Hypertonic medium was the most toxic, so a maximum of 10-min exposure time was used. DAPI dye was used to positively identify dead cells, and positively stained cells had similar *D* values as those not permeable to DAPI, indicating temporary permeabilization rather than death^60^. Notably, some cells observed by TEM demonstrated membrane damage, but major cellular structures remained intact. Further evaluation of cellular effects such as changes in gene expression were not pursued. The QDs were stable in the loading medium for at least the duration of the loading step (Supplementary Fig. 19). For passive uptake of QDs, CHO cells were seeded at a density of 20,000 cells/cm^2^ in LabTek chambers 24 h before the addition of 10 nM pPEG QDs in complete medium without phenol red. Uptake was assessed by microscopy after 24 h, immediately after washing.

### Fluorescence microscopy

Fluorescence imaging was performed using wide-field illumination on a Zeiss Axio Observer.Z1 inverted microscope with a 100×1.45 NA alpha Plan-Fluar oil immersion objective. QD images in cells were acquired with HILO excitation, with a 488 nm 100 mW optically pumped semiconductor laser with 15% laser power at the optimized HILO angles for our system (~60° from normal). Excitation light was filtered using a 482/18 laser-line bandpass filter (Semrock), and emission light was filtered using a 600/37 bandpass filter (Semrock). Images were acquired using a Photometrics eXcelon Evolve 512 EMCCD using Zeiss Zen software. The QDs were imaged at 19.6 frames per second, 40 min after equilibration at 37 °C with a focal plane set at the largest cross-section of the cell, a few microns above the center of the nucleus.

### Single-particle tracking and diffusion analysis

Single QD videos were analyzed by SPT using the MATLAB u-track software package developed by Jaqaman et al.^35^ to determine the centroid pixel positions (*x*_o_,*y*_o_) for trajectories at each time point *t*. Custom MATLAB scripts were used to calculate the mean squared displacement (MSD) versus time increment (*τ*) curves for each particle trajectory, and were fit to a model of anomalous diffusion in Eq. (1)^36,37,61,62^. For tracks longer than 100 frames, the curves were fit for the first 10 time increments, whereas ¼ of the track length was fit for shorter tracks. Tracks shorter than 10 frames were discarded (track length distributions are shown in Supplementary Fig. 20). These lengths were selected based on the recommendations of Kepten and colleagues for tracks in the regime of strong subdiffusion to weak superdiffusion (*α* = 0.3–1.3), with low localization error^63^. We evaluated the impact of the localization error on *D* versus *α* heat maps (Supplementary Fig. 21) and evaluated the impact of time increment span for curve fitting (Supplementary Fig. 22), and found that both had fairly small impacts on the absolute mobile fractions. Curve fits were filtered based on the calculated error of the fitting parameters, with error tolerances of 0.05 μm^2^/s for *D* and 0.15 for *α*.

### Optical analysis

The brightness of the particle, *B*(*t*), located at the centroid position (*x*_o_,*y*_o_), was calculated by averaging the intensities of a 3 × 3 pixel area at each detected particle centroid, according to the following equation:

3 $$B\left( t \right) = \frac{1}{9}\mathop {\sum }\limits_{x = [x_0] - 1}^{[x_0] + 1} \mathop {\sum }\limits_{y = [y_0] - 1}^{[y_0] + 1} I(x,y,t)$$

where [*x*_*o*_] and [*y*_*o*_] are the centroid positions rounded to the nearest pixel integer, and *I*(*x,y,t*) is the intensity of a pixel as a function of position and time. To analyze the differing levels of clustering or aggregation, the maximum relative brightness of a particle, $B_{{\mathrm{rel}}}^{{\mathrm{max}}}$, was calculated as the mode of the top 6% of the *B*(*t*) distribution of its complete trajectory:

4 $$B_{{\mathrm{rel}}}^{{\mathrm{max}}} = Mo[B\left( {0.94 \le P(B) \le 1} \right)]$$

where *P*(*B*) is the probability distribution of brightness values from an intensity time trace, *B*(*t*). Because QDs randomly fluctuate between on and off states (blinking), only the brightest state was considered in calculating $B_{{\mathrm{rel}}}^{{\mathrm{max}}}$, and we previously determined that the single-QD on-state brightness is homogeneous among uniform QDs with this composition (Fig. 1f)^38^.

The mean brightness of a single QD for a single cell, $B_{{\mathrm{rel}}}^1$, was then calculated as the center brightness value of the first peak in a $B_{{\mathrm{rel}}}^{{\mathrm{max}}}$ distribution, using the first derivative of the $B_{{\mathrm{rel}}}^{{\mathrm{max}}}$ distribution, defined as:

5 $$B_{{\mathrm{rel}}}^1 = \left( {\frac{{{\mathrm{d}}N}}{{{\mathrm{d}}B_{{\mathrm{rel}}}^{{\mathrm{max}}}}}} \right)_{0,1}$$

where *N* is the number of trajectories as a function of $B_{{\mathrm{rel}}}^{{\mathrm{max}}}$. This value, together with $B_{{\mathrm{rel}}}^{{\mathrm{max}}}$ for each particle, was used to calculate the number of QDs per cluster (*n*_QD_) using Eq. (2), the fraction of single, unclustered QDs (*f*_1_), and the total number of QDs per cell (*N*_cell_).

*f*_1_ was calculated by integrating the area under the single-QD peak in the plot of *N*, the number of trajectories, as a function of $B_{{\mathrm{rel}}}^{{\mathrm{max}}}$. Since our previous findings have shown that single QDs have Gaussian brightness distributions^38^, we assumed the single-QD peak was Gaussian. $B_{{\mathrm{rel}}}^1$ was set as the mean of the single-QD peak, and we calculated the half-width of the single-QD peak as the difference between $B_{{\mathrm{rel}}}^1$ and $B_{{\mathrm{rel}}}^0$, where $B_{{\mathrm{rel}}}^0$ is the minimum brightness value of the $B_{{\mathrm{rel}}}^{{\mathrm{max}}}$ distribution. With the assumption that the distribution was symmetrical, the upper limit of the single-QD peak was defined by the mean of the distribution, $B_{{\mathrm{rel}}}^1$, plus the half-width,$B_{{\mathrm{rel}}}^1$ – $B_{{\mathrm{rel}}}^0$. After setting the upper and lower bounds of the single-QD peak, we calculated *f*_1_ by dividing the number of trajectories between these two bounds (i.e., the number of single QDs) by the total number of trajectories. This calculation is represented by the following equation:

6 $$f_1 = \frac{{\mathop {\int }\nolimits_{B_{{\mathrm{rel}}}^0}^{B_{{\mathrm{rel}}}^1 + (B_{{\mathrm{rel}}}^1 - B_{{\mathrm{rel}}}^0)} N\left( {B_{{\mathrm{rel}}}^{{\mathrm{max}}}} \right){\mathrm{d}}B_{{\mathrm{rel}}}^{{\mathrm{max}}}}}{{\mathop {\int }\nolimits_0^\infty N\left( {B_{{\mathrm{rel}}}^{{\mathrm{max}}}} \right){\mathrm{d}}B_{{\mathrm{rel}}}^{{\mathrm{max}}}}}$$

where the numerator is the number of single QDs, as described above, and the denominator is the total number of trajectories. This method of assigning an upper brightness threshold for the single-QD peak has the possibility of misidentifying some non-single trajectories as single. Using the assumption that the single-QD distribution is symmetrical, we calculated the frequency of incorrect single-QD identification (false positives), shown in Supplementary Fig. 23. All data sets with a false positive rate of 25% or greater were excluded from further analysis.

$B_{{\mathrm{rel}}}^1$ and *N* (the number of trajectories as a function of $B_{{\mathrm{rel}}}^{{\mathrm{max}}}$) were used to calculate the number of QDs per cell, *N*_cell_. Dividing $B_{{\mathrm{rel}}}^{{\mathrm{max}}}$ by $B_{{\mathrm{rel}}}^1$ converts the units of $B_{{\mathrm{rel}}}^{{\mathrm{max}}}$from brightness to QD number, after which *N* values are summed to calculate the total number of QDs in a cell, *N*_cell_:

7 $$N_{{\mathrm{cell}}} = \frac{1}{{B_{{\mathrm{rel}}}^1}}\mathop {\sum }\limits_0^{{\infty }} N\left( {B_{{\mathrm{rel}}}^{{\mathrm{max}}}} \right)$$

Note that the actual number of QDs per cell is greater than what is measured due to the limited focal volume, and the actual physical value is estimated to be approximately twice this value for the full volume of the cell. This twofold difference is accounted for in the theoretical value of *N*_cell_ described in the “Uptake efficiency” section.

### Simulation analysis

Trajectories were simulated using the MATLAB *wfbm* function, which generates a fractional Brownian motion trajectory with specified *α*, when normalized by standard deviation. Trajectories were scaled by *D*, and a series of normally distributed measurement errors were added to each position in each dimension^63^. Measurement errors derived from a distribution with zero mean and a standard deviation derived from the mean experimental localization error from the SPT analysis for each QD coating class. Track lengths were truncated to match the distribution of experimentally measured track lengths for each QD coating (Supplementary Fig. 20). These simulated trajectories were analyzed using the diffusion analysis method described above.

### Code availability

The Matlab codes used in this study are available from the corresponding author on reasonable request.

### Data availability

The data that support the findings of this study are available from the corresponding author on reasonable request.

## Electronic supplementary material

Supplementary Information

Description of Additional Supplementary Files

Supplementary Movie 1

Supplementary Movie 2

Supplementary Movie 3

Supplementary Movie 4

Supplementary Movie 5

Supplementary Movie 6

Supplementary Movie 7
