# Supplementary material for: Single quantum dot tracking reveals the impact of nanoparticle surface on intracellular state
Source: Nat Commun. 2018 May 8;9:1830. doi: 10.1038/s41467-018-04185-w (PMC5940692; doi:10.1038/s41467-018-04185-w)
Supplement: Supplementary file 1 — Supplementary Information [file 41467_2018_4185_MOESM1_ESM.pdf]

Supplementary Figures

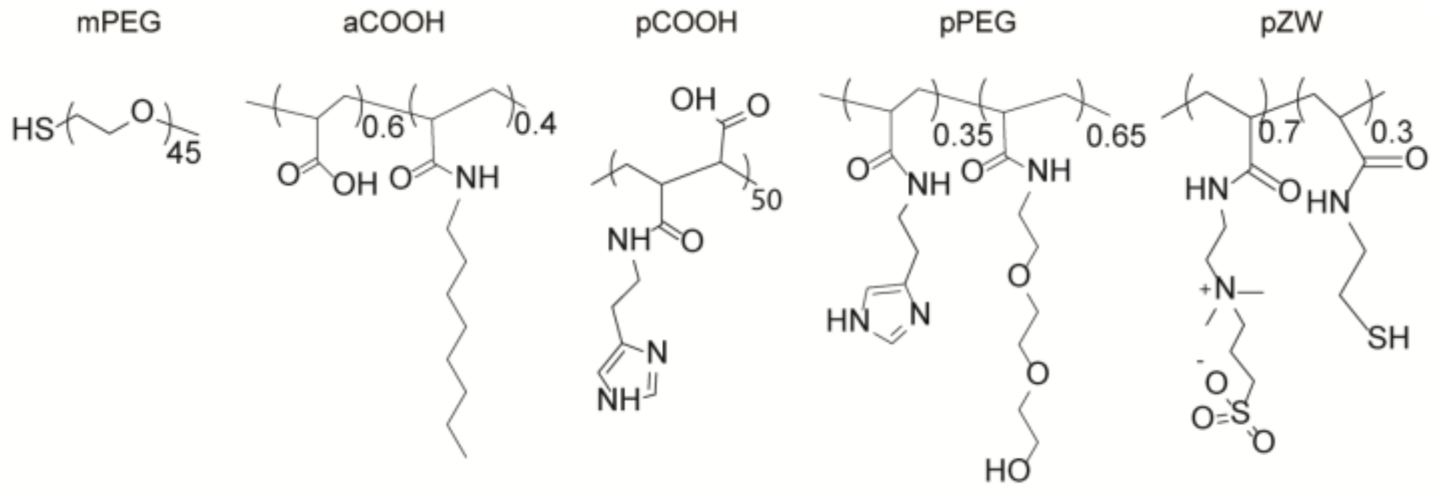

Supplementary Figure 1. Structures of polymeric QD coatings used in this work.

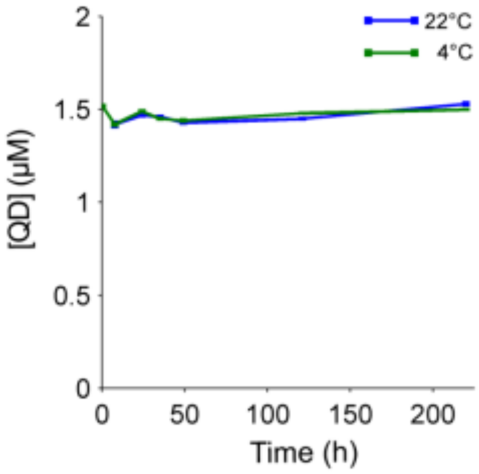

Supplementary Figure 2. Stability of mPEG-QDs in 50 mM borate buffer. Stability was measured by centrifuging at 7000 x *g* for 10 minutes to remove aggregates that may have formed due to coating instability. Concentrations were determined by measuring absorbance values at 350 nm. n = 3 for both temperature conditions. Error bars indicate s.e.m.

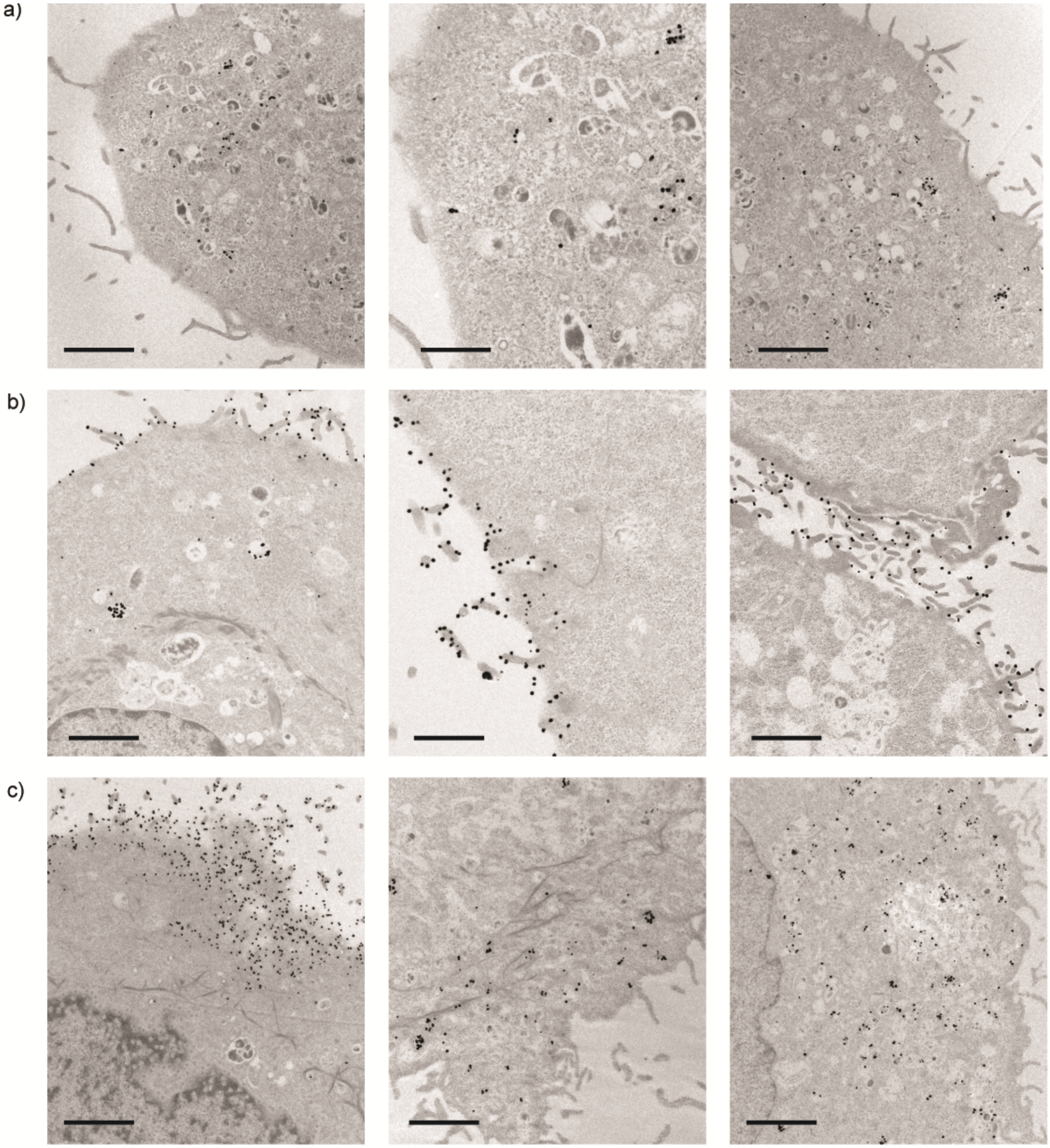

Supplementary Figure 3. TEM images of mPEG-QD delivery to A431 cells. QDs were developed with silver to increase nanoparticle contrast.[1] (a) Cells exposed to QDs in complete medium show QDs primarily trapped in endosomes. (b) Cells exposed to QDs in hypertonic loading buffer, showing QDs trapped in pinosomes, adhering to the cell membrane, and localized in gaps between cells. (c) Cells exposed to QDs in hypertonic loading buffer, followed by addition of hypotonic lysis buffer, showing QDs in the cytoplasm near the cell membrane and further inside the cell. All scale bars indicate 2 μm.

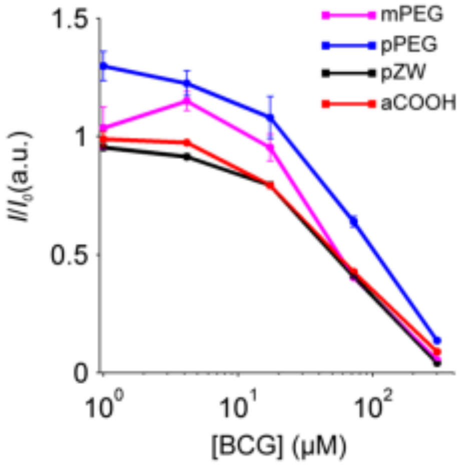

Supplementary Figure 4. Fluorescence quenching of QDs with bromocresol green (BCG). QDs were coated with mPEG, pPEG, pZW, or aCOOH. n = 3 for all QD coatings; error bars indicate s.e.m.

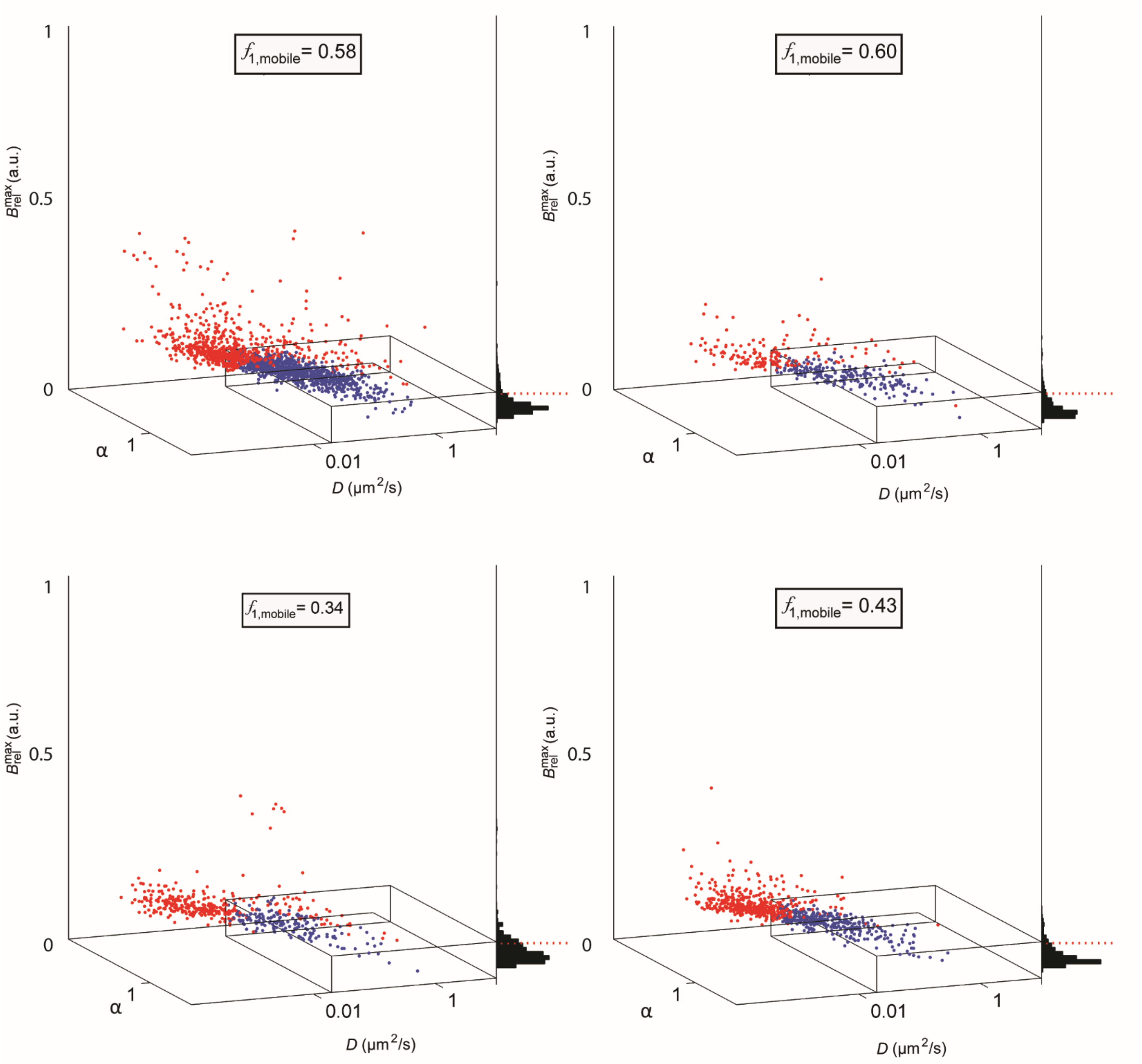

Supplementary Figure 5. 3D plots of D versus α versus B_{rel}^{max} for pPEG QDs. Heterogeneity of brightness distributions and QD clustering is apparent between different cells.

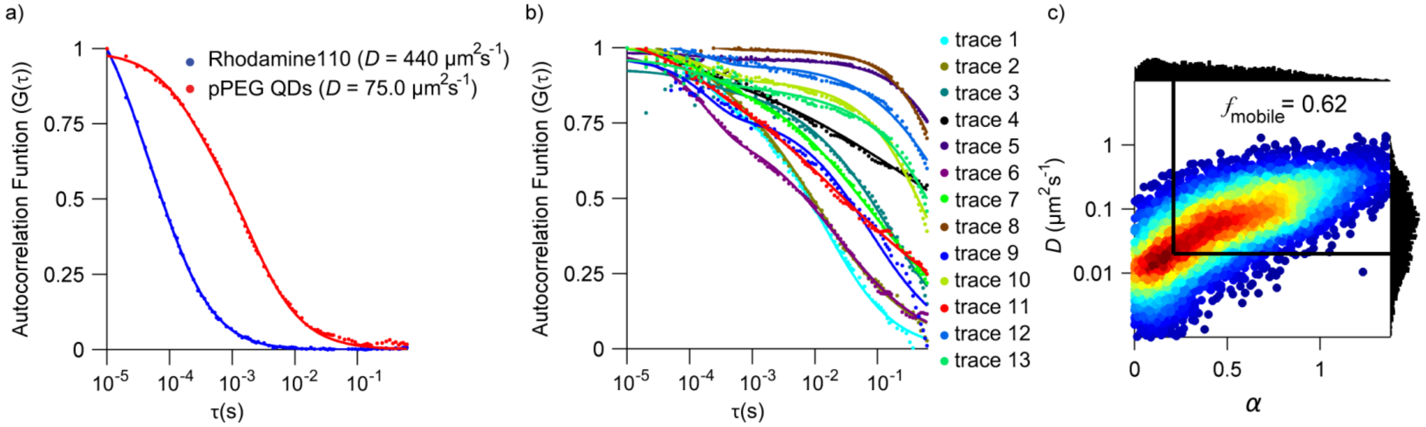

Supplementary Figure 6. Fluorescence correlation spectroscopy (FCS) data. (a) FCS autocorrelation curves for Rhodamine 110 and pPEG-QDs in aqueous buffer fit to a Brownian diffusion model. (b) FCS autocorrelation curves for pPEG-QDs delivered by OPL to HeLa cells, fit to the multi-component anomalous diffusion model shown in Supplementary Equation 3. n = 13 samples. Fit parameters for the components of each trace are shown in Supplementary Table 2. (c) Heat map of *D* versus *α* for pPEG-coated QDs delivered to HeLa cells, calculated by SPT data. n = 9 cells.

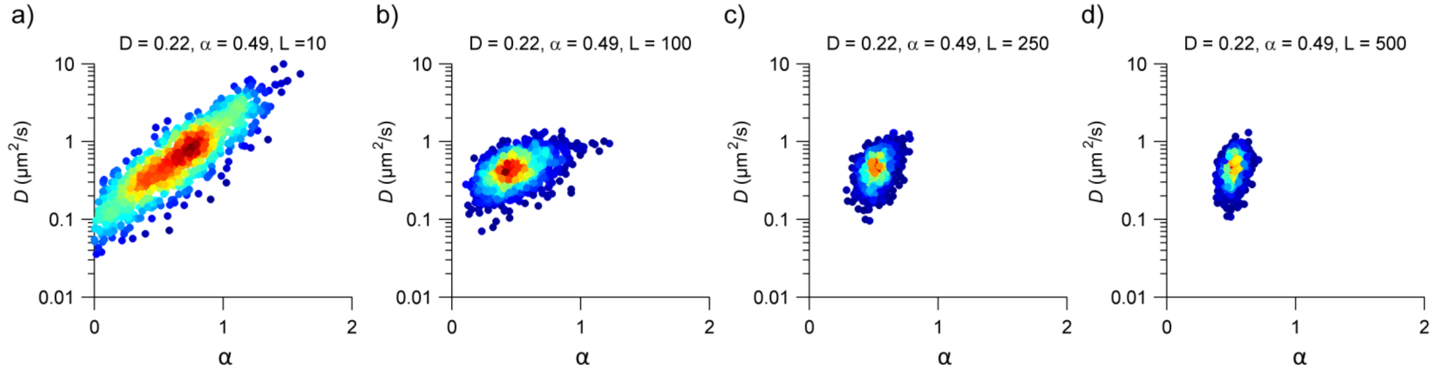

Supplementary Figure 7. Simulation analysis of track length impact. Heat map of *D* versus *α* for 1000 trajectories with D = 0.22 µm^2 s^{-1} and α = 0.49 (average values for pZW-QDs) with fixed track lengths of (a) 10, (b) 100, (c) 250, and (d) 500 frames. Simulated localization error values were derived from experimentally measured average values from pZW-QDs.

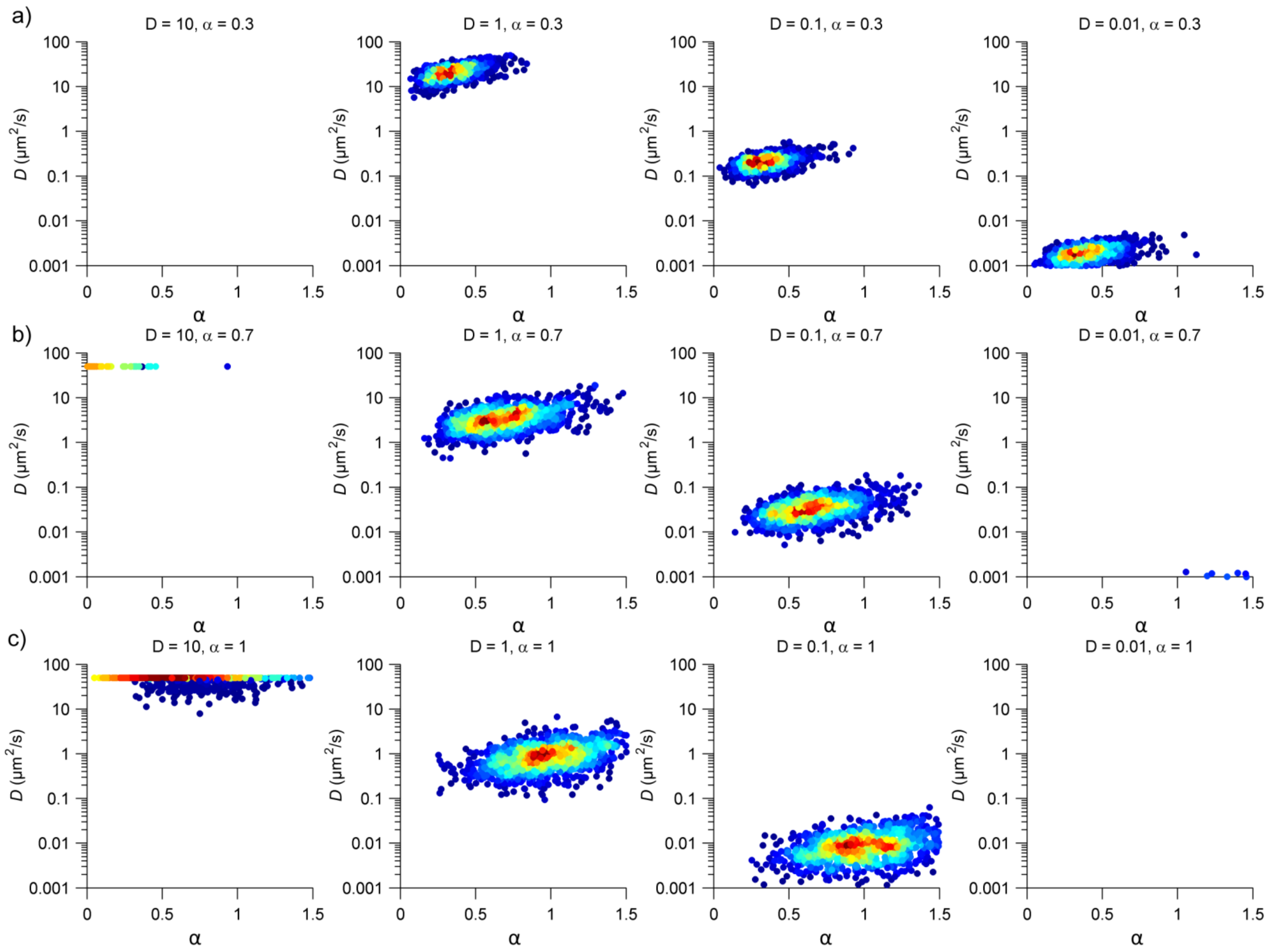

Supplementary Figure 8. Simulation analysis of the impact of *D* and *α*. For each plot, a fixed track length (L = 1000) was used with 1000 trajectories. Heat maps show *D* versus *α* with *D* ranging from 0.01 to 10 μm^2 s^{-1} in each row and *α* of (a) 0.3, (b) 0.7, and (c) 1. Simulated localization error values were derived from experimentally measured values from pZW QDs.

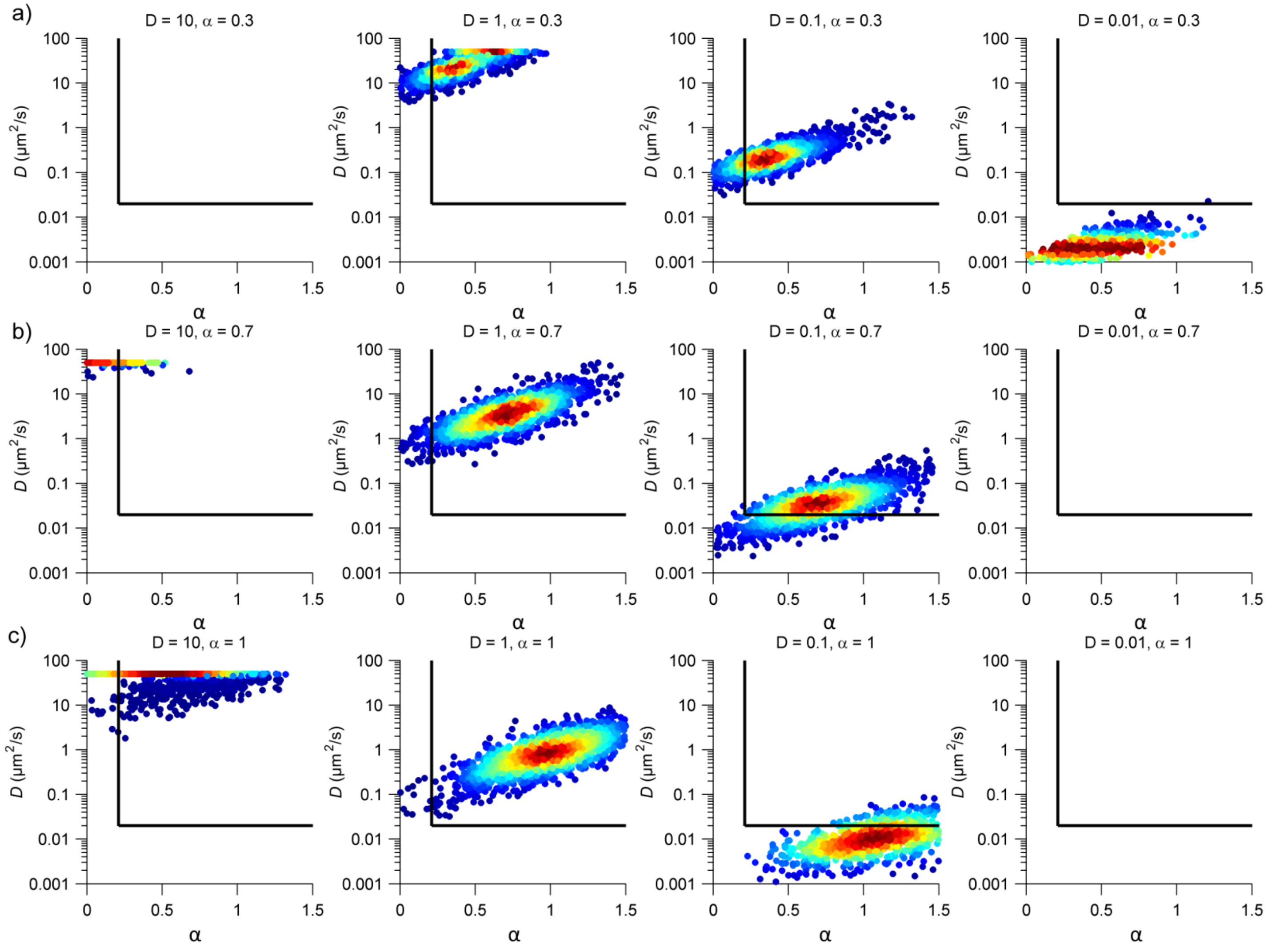

Supplementary Figure 9. Simulation analysis of the impact of *D* and *α*. Track length distributions were derived from empirical results from pZW QDs (see Supplementary Figure 18) with 1610 trajectories. Heat maps show *D* versus *α* with *D* ranging from 0.01 to 10 μm^2 s^{-1} in each column and *α* = (a) 0.3, (b) 0.7, and (c) 1. Simulated localization error values were derived from experimentally measured values from pZW QDs.

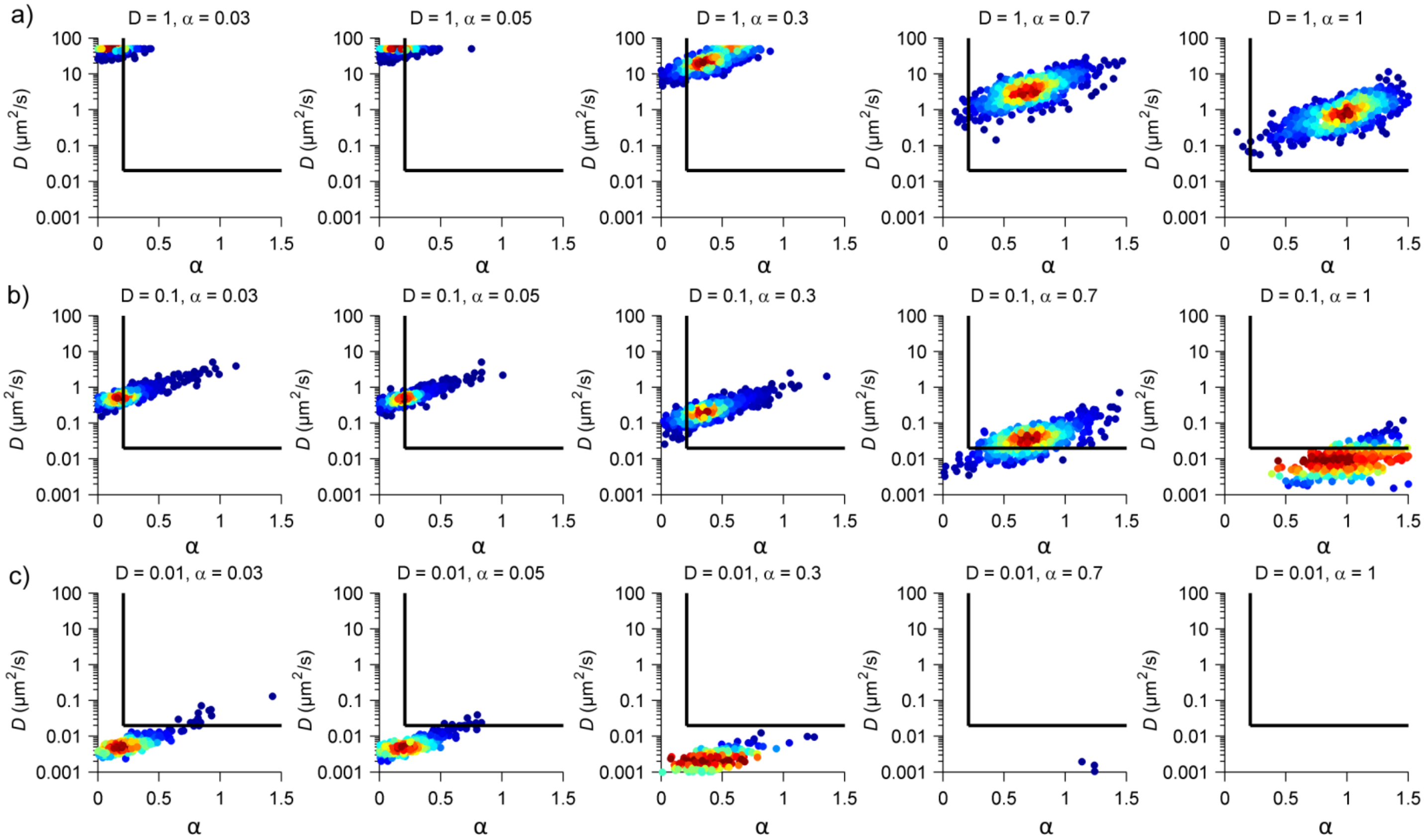

Supplementary Figure 10. Simulation analysis of the impact of *D* and *α*. Track length distributions were derived from empirical results from mPEG QDs (see Supplementary Figure 13) with 1610 trajectories. Heat maps show *D* versus *α* with *α* ranging from 0.03 to 1 in each column and *D* of (a) 1, (b) 0.1, and (c) 0.01 $\mu m^2\ s^{-1}$. Simulated localization error values were derived from experimentally measured values from mPEG QDs.

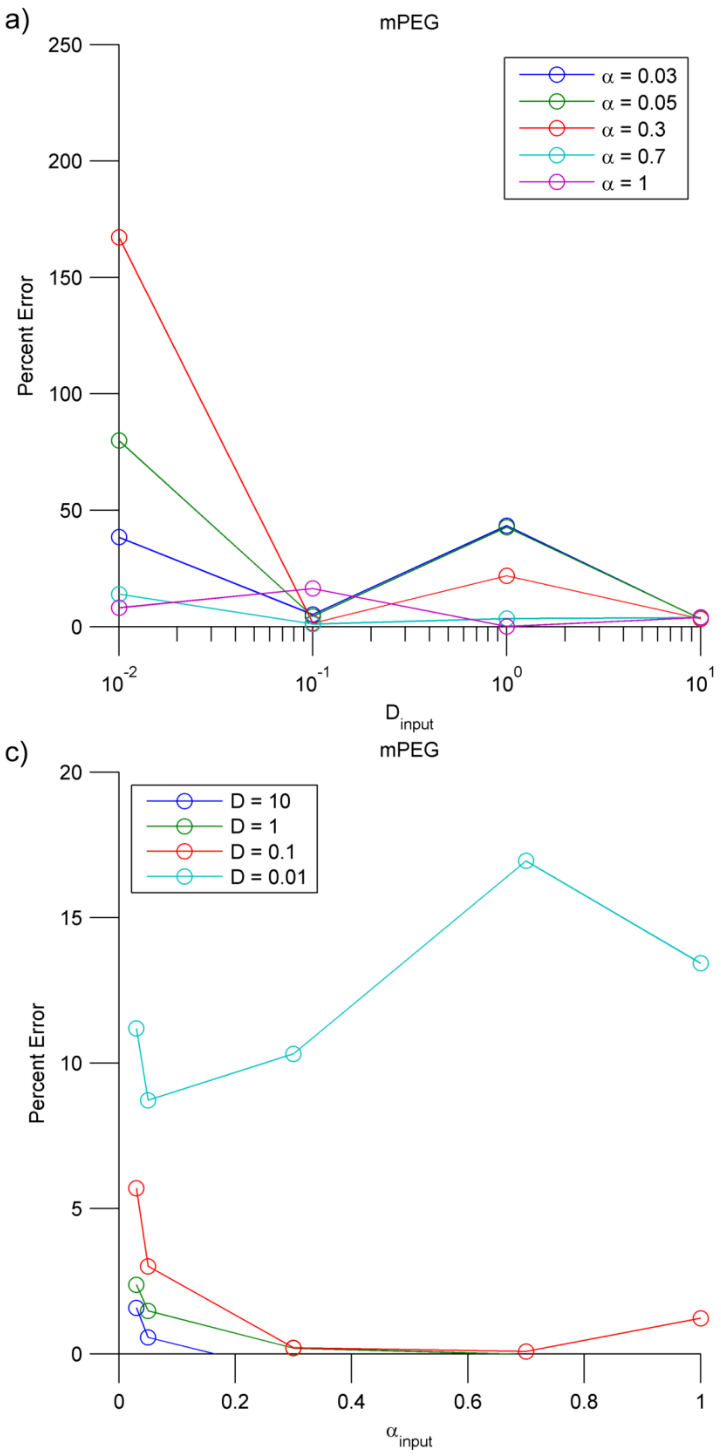

Supplementary Figure 11. Error in D and α measured from simulation results. (a,b) Plots show percent difference between the calculated average D and input D for track length distributions and localization error values derived from empirical data from (a) mPEG-QDs and (b) pZW-QDs. (c,d) Plots show percent difference between the calculated average α value and input α values for track length distributions and localization error values derived from empirical data from (c) mPEG-QDs and (d) pZW-QDs.

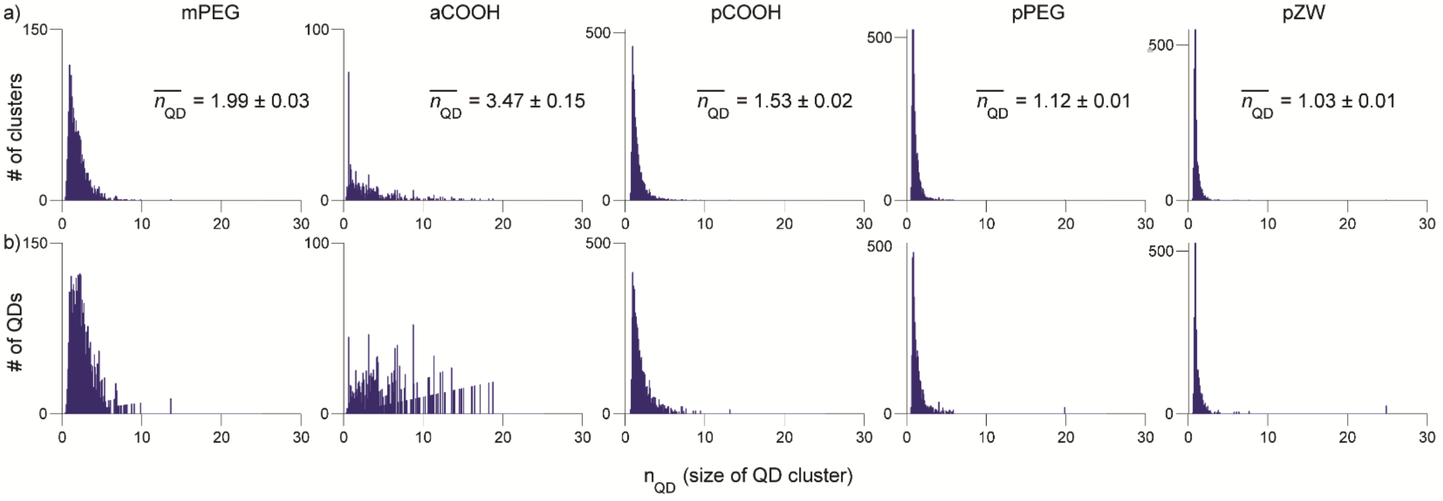

Supplementary Figure 12. Comparison of n_{QD} binning method for QD coating comparison. (a) Histograms of n_{QD} (number of QDs per cluster detected) and (b) weighted n_{QD} (number of QDs in a cluster of a given size) for QD coating comparison.

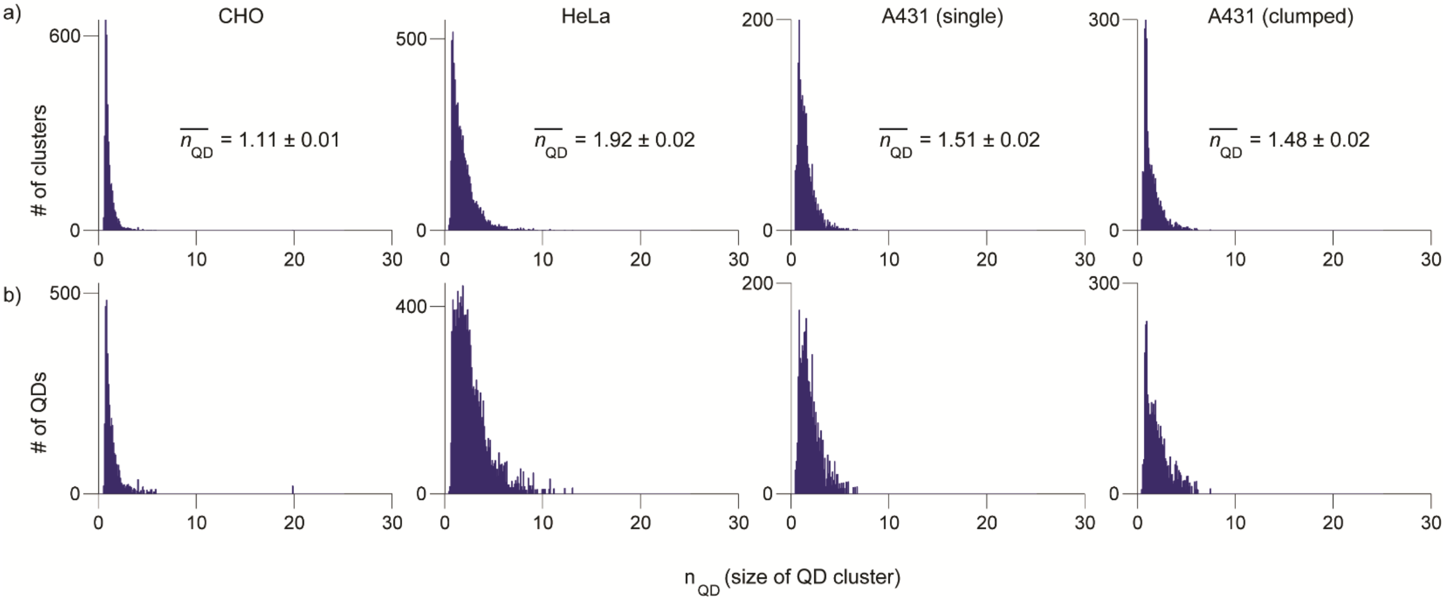

Supplementary Figure 13. Comparison of n_{QD} binning method for cell type comparison. (a) Histograms of n_{QD} (number of QDs per cluster detected) and (b) weighted n_{QD} (number of QDs in a cluster of a given size) for cell type comparison.

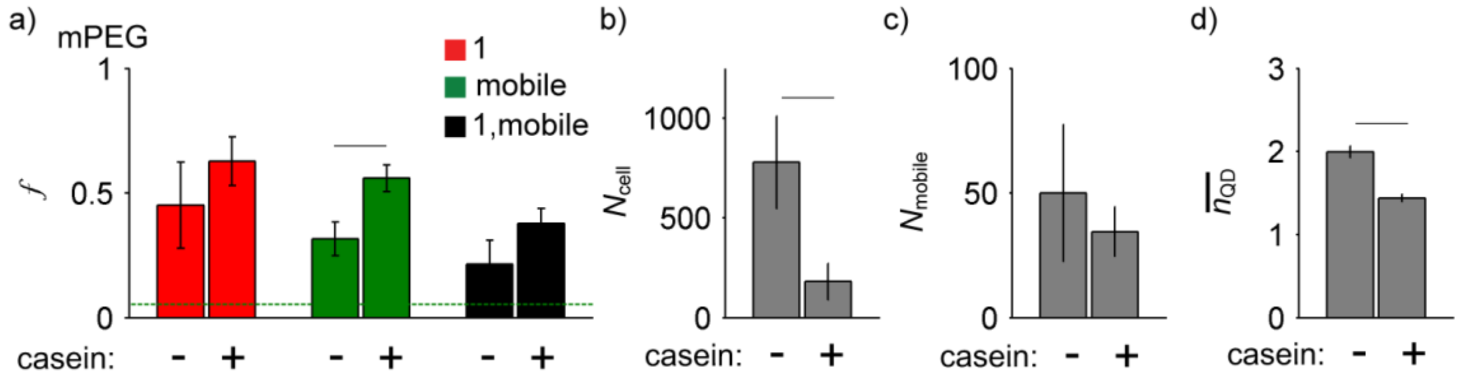

Supplementary Figure 14. Mobility, delivery, and clustering metrics for QDs. QDs coated with mPEG were loaded into CHO cells, with and without casein added to block nonspecific binding. (a) Aggregated data are plotted to show f_1, f_{mobile}, and $f_{1,mobile}$ for mPEG-QDs with and without casein blocking. The green dashed line in indicates the lower limit for f_{mobile} (0.040) which corresponds to the localization error. (b) Number of internalized QDs per cell for mPEG-QDs with and without blocking for 10 nM loading concentration. The sharp decrease in N_{cell} for the mPEG QDs with the addition of casein to block nonspecific adsorption demonstrates that nonspecific interaction with the cell surface is likely to be the underlying mechanism for the high level of intracellular delivery for mPEG-coated QDs. (c) Total number of mobile mPEG-QDs per cell for each condition, showing that blocking does not significantly alter the number of mobile QDs per cell. (d) Effect of casein blocking on mPEG-QD clustering. The decrease in $\overline{n_{QD}}$ for mPEG with casein similarly demonstrates that nonspecific adsorption is associated higher levels of QD clustering. Horizontal black lines indicate $p < 0.05$. For all experimental conditions, n = 6 cells per group. All error bars indicate s.e.m.

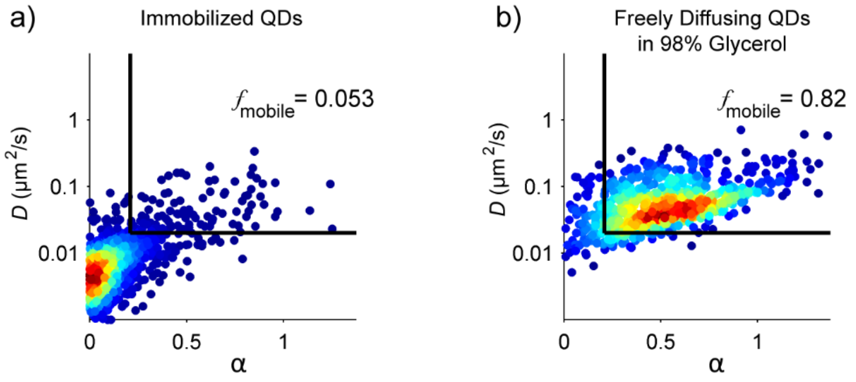

Supplementary Figure 15. Density plots of D versus α. Data indicate (a) immobilized QDs (f_{mobile} = 0.053) and (b) freely diffusing QDs in 98% glycerol (f_{mobile} = 0.82). These plots demonstrate localization error as well signatures of freely diffusing particles.

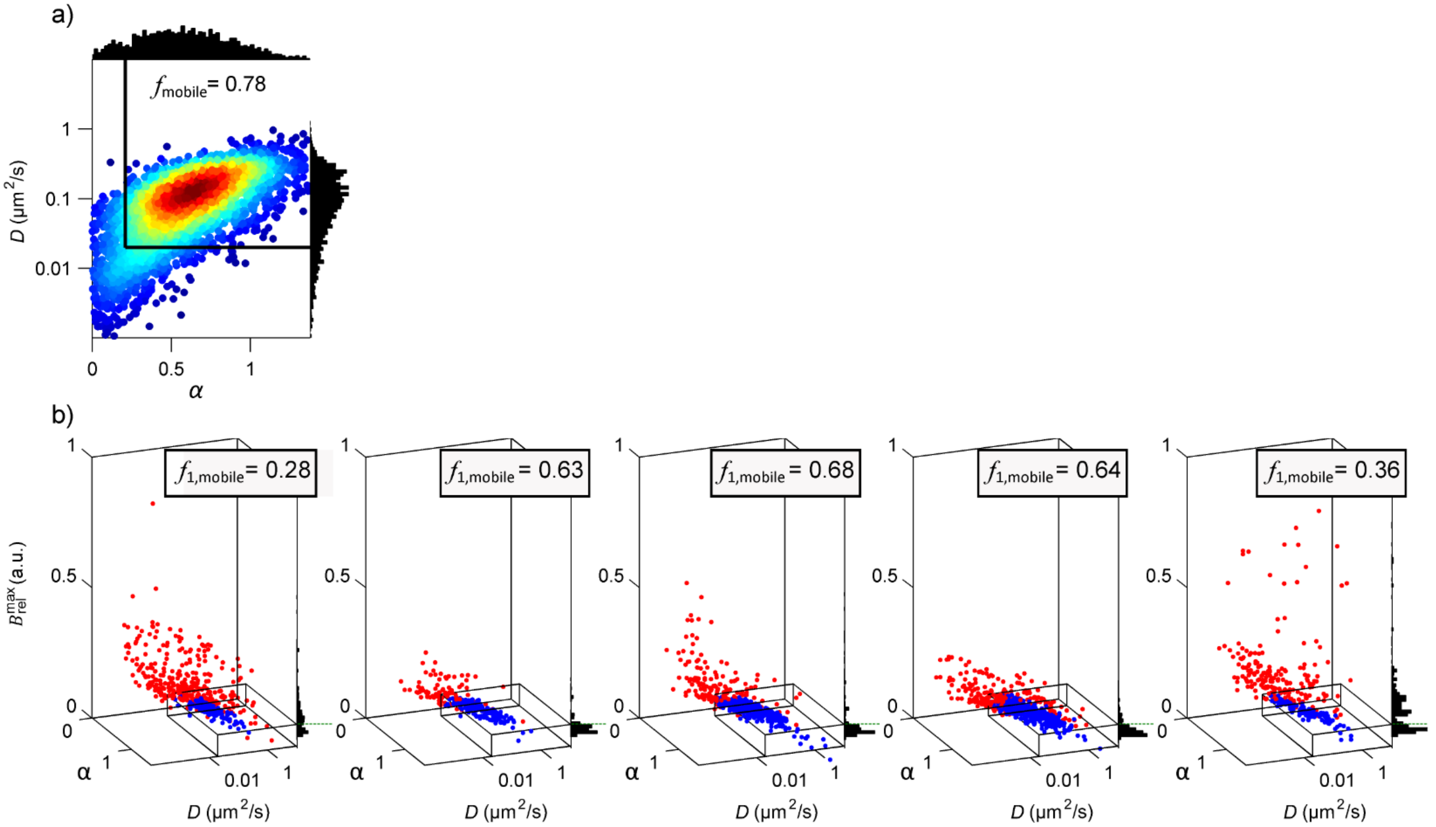

Supplementary Figure 16. Diffusion and optical analysis after Lipofectamine delivery. QDs coated with pPEG were delivered to CHO cells. (a) Heat map of D versus α; n = 8 cells. (b) Representative 3D plots of D versus α versus B_{rel}^{max} showing heterogeneity of brightness distributions and QD clustering between different cells.

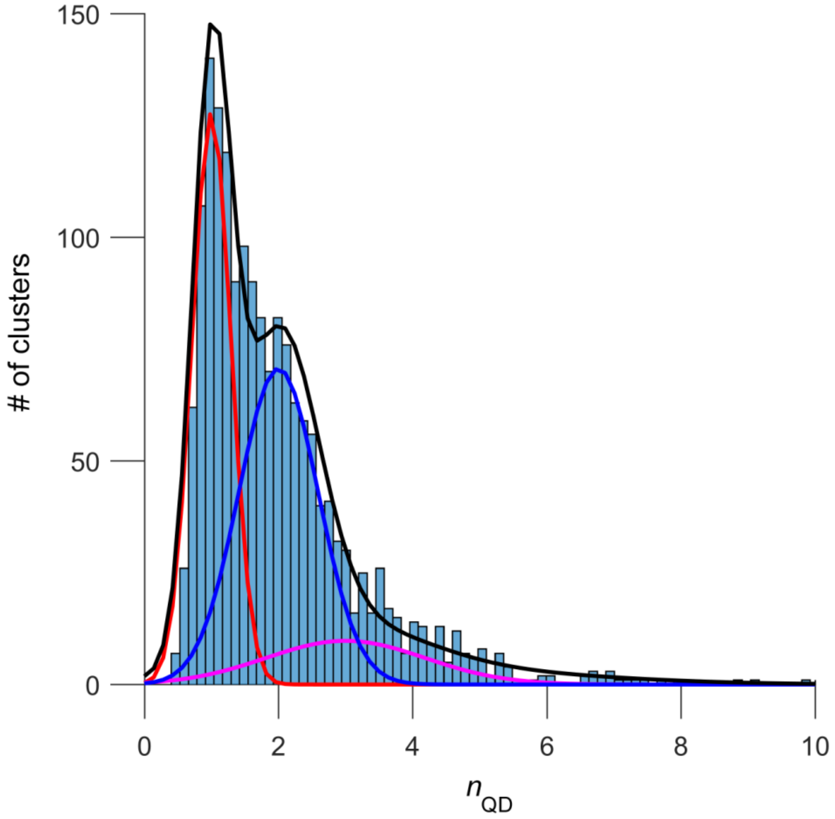

Supplementary Figure 17. Histogram of n_{QD} values. Data show mPEG-coated QDs with superimposed Gaussian fits demonstrating the quantized brightness distributions apparent with more highly clustered QDs.

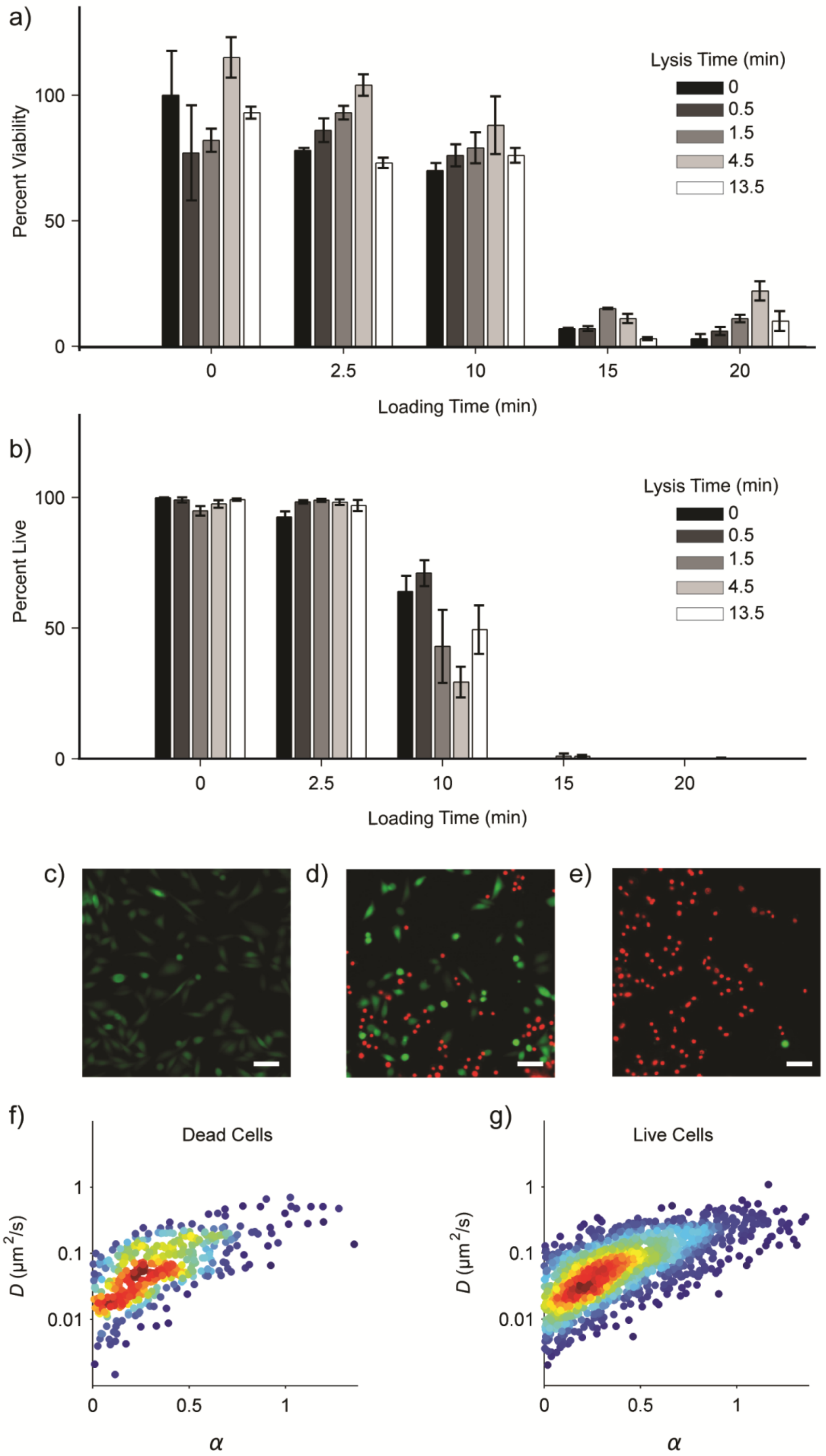

Supplementary Figure 18. Live/dead and viability after loading and lysis treatments. (a) CCK-8 viability assay for the indicated loading and lysis times. (b) Live/dead analysis using Calcein AM and EtH-1 for the indicated loading and lysis times. n = 3 for all experimental conditions. Error bars in panels (a) and (b) indicate s.e.m. Sample images depict loading times of (c) 2.5, (d) 10, and (e) 15 min, all with 1.5 min lysis. All scale bars indicate 50 μm. (f, g) Density plots of D versus α for pPEG-QDs loaded in CHO cells for cells that are (f) dead or (g) live, determined by DAPI-based membrane permeabilization, with n = 9 and 26 cells, respectively.

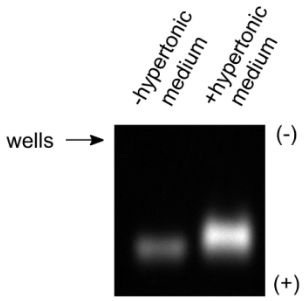

Supplementary Figure 19. Assessment of pPEG-coated QDs in hypertonic loading medium. pPEG-QDs (40 nM) were incubated in 50 mM borate buffer or the hypertonic loading medium composed of PEG, sucrose, and incomplete DMEM, 10 minutes, after which the uniformity of the QDs was assessed by gel electrophoresis in a hybrid acrylamide/agarose gel (120V for 20 minutes at 4°C). A uniform band was observed under both conditions, indicating that the QDs are stable for at least the duration of the delivery step. The difference in migration distance is due to the high viscosity of the hypertonic medium, which was loaded neat into the gel lane.

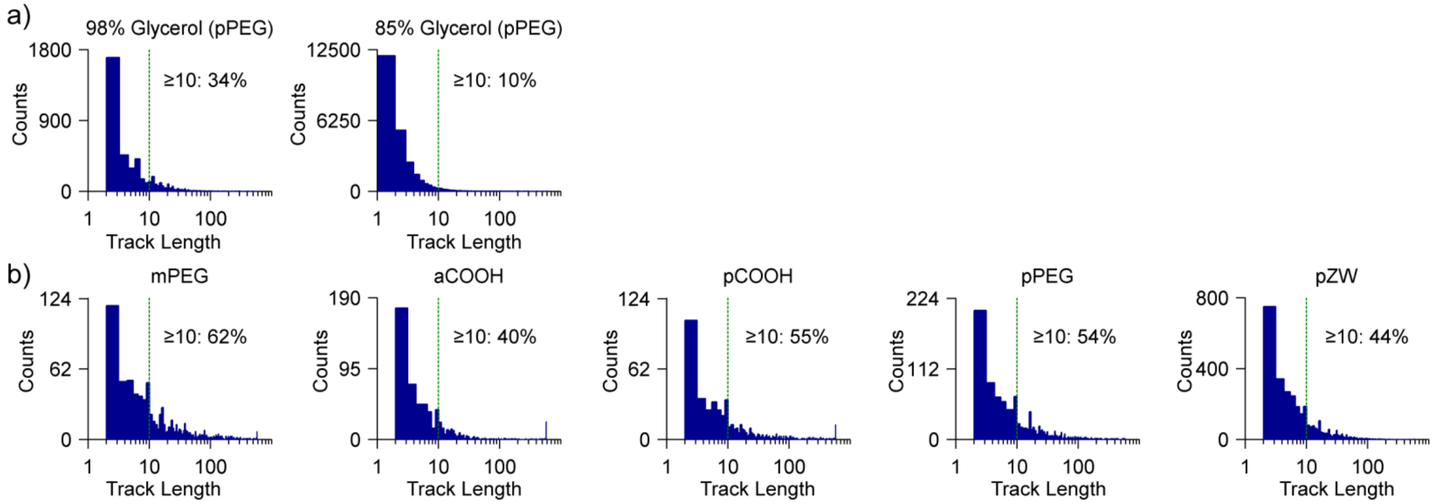

Supplementary Figure 20. Track length histograms of QDs in glycerol solution or cells. (a) Track length distributions of QDs diffusing in aqueous solutions of glycerol with indicated glycerol weight percentage. The percentage of tracks longer than 10 frames is noted for both solutions. The reduced number of >10-frame tracks in the 85% glycerol solution sets a maximum value of measurable *D* by 2D SPT. (b) Track length distributions for QDs with the 5 different coatings in CHO cells. Note that the pZW-QD and pPEG-QD samples have higher measured *D* compared with QDs diffusing in 98% glycerol because they demonstrate a larger percentage of tracks longer than 10 frames, due to z-axis confinement. Data correspond to the same as that of Figure 4a-b.

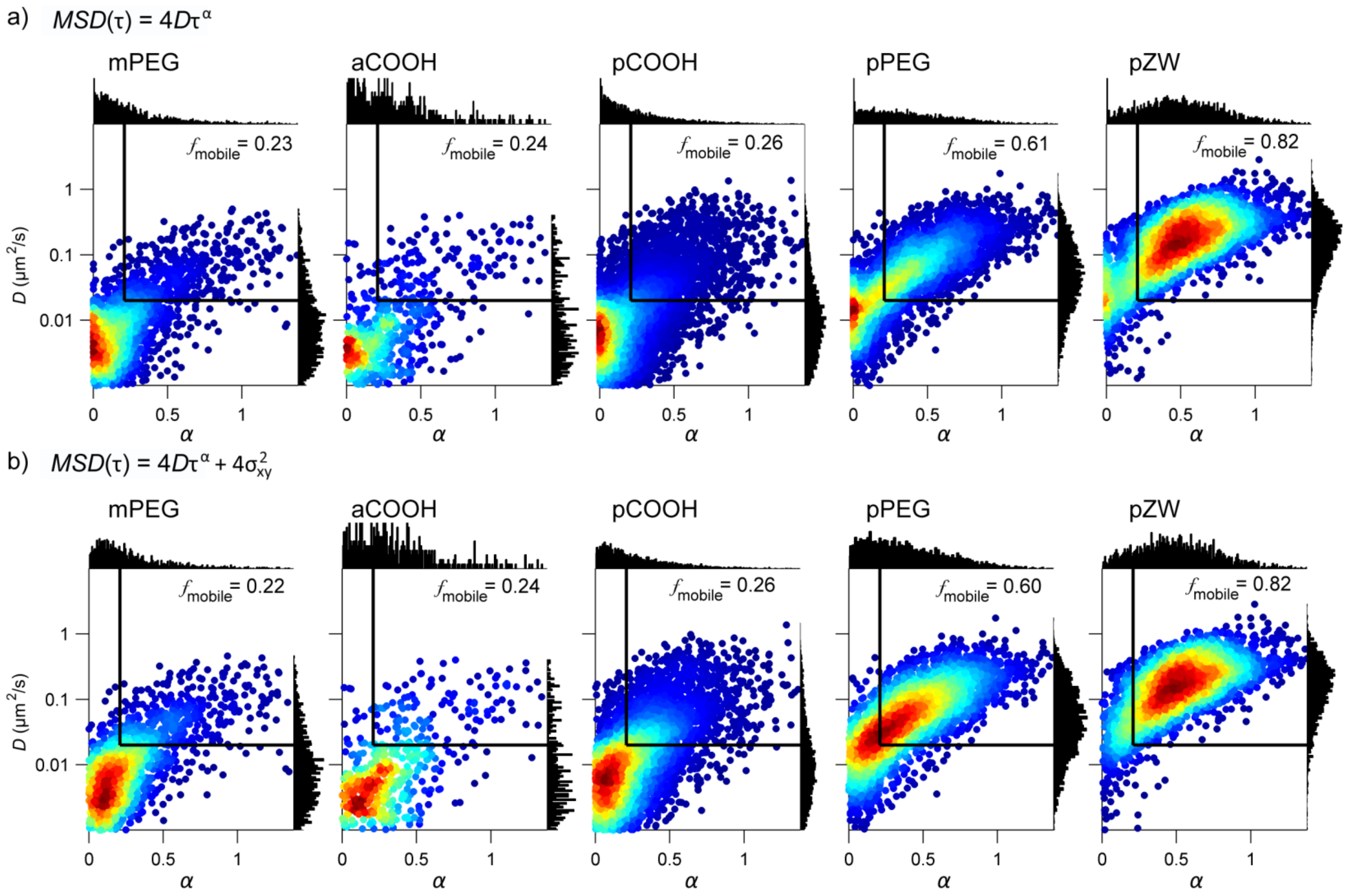

Supplementary Figure 21. Localization error correction. Heat maps compare *D* versus *α* plots for intracellular QDs with 5 coatings, using the same data from Figure 4a in the main text. (a) Data derived from *MSD* fitting to the anomalous diffusion model without the localization error correction factor. (b) Data derived from *MSD* fitting to the anomalous diffusion model with the localization error correction factor.

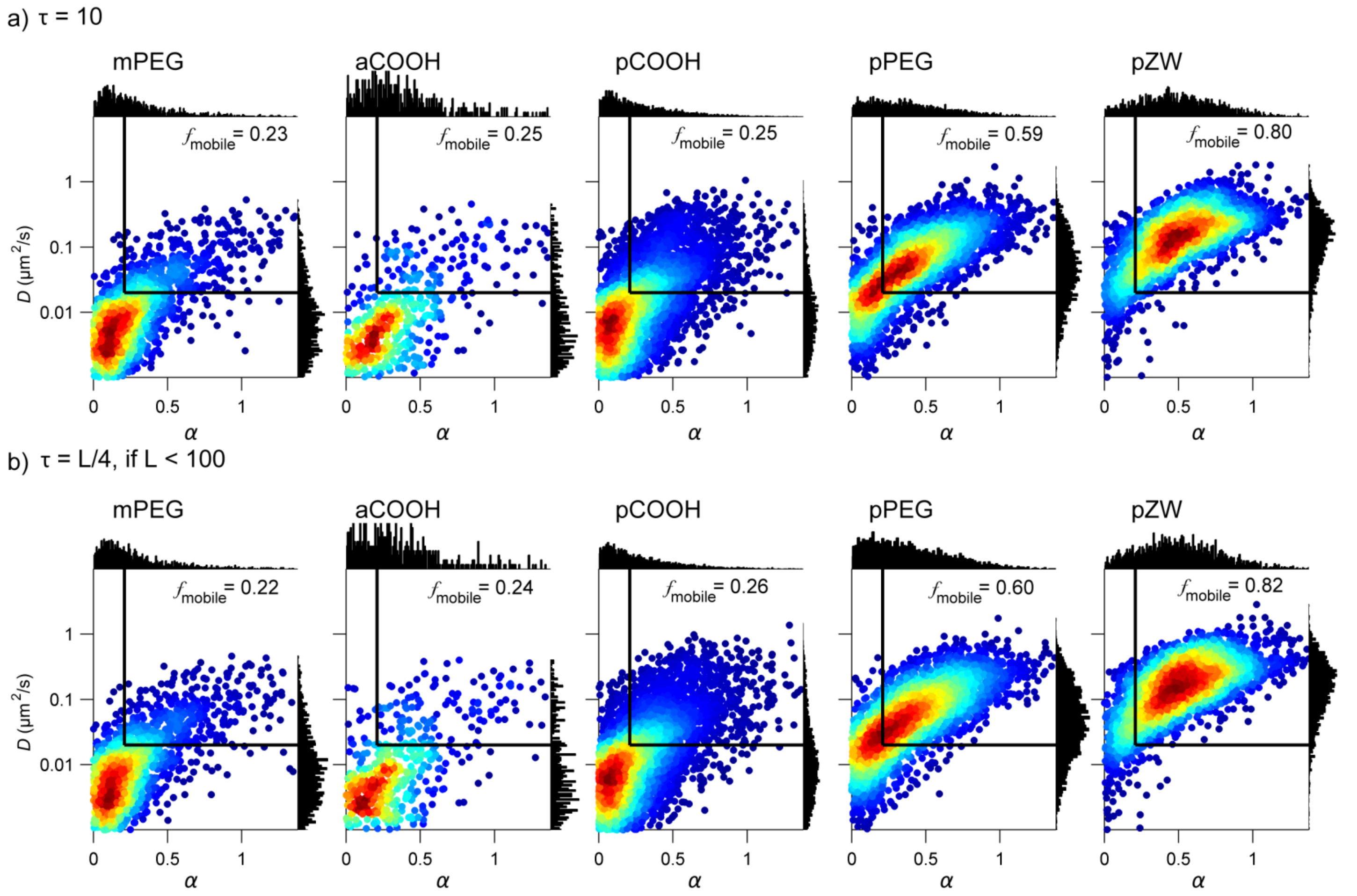

Supplementary Figure 22. MSD time lag fitting analysis. Heat maps compare *D* versus *α* for intracellular QDs with 5 coatings, using the same data from Figure 4a in the main text. (a) Data derived from *MSD* fitting to the anomalous diffusion model with localization error correction factor, 10 time lags, τ, for all trajectories. (b) Data derived from *MSD* fitting to the anomalous diffusion model with localization error correction factor, using *L*/4 time lags, where *L* is the trajectory length if the trajectory was shorter than 100 frames. For trajectories longer than 100 frames, 10 time lags were used.

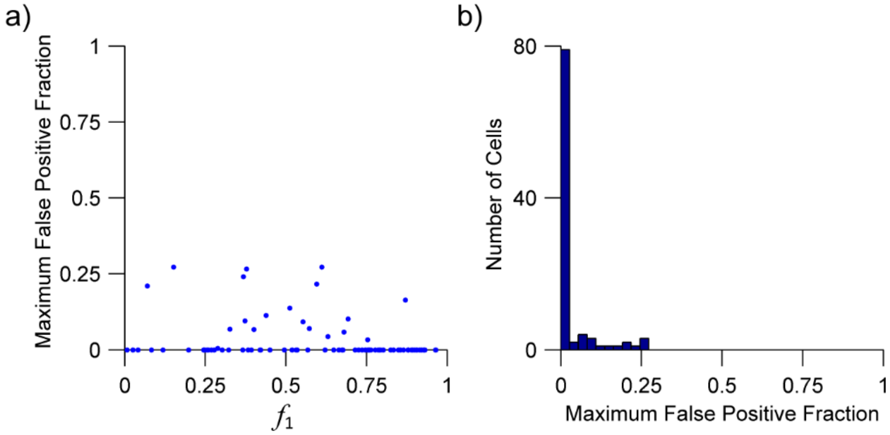

Supplementary Figure 23. f_1 error analysis. In order to assign a state of single or non-single to every trajectory, an upper brightness threshold was set based on the assumed width of the single-QD peak, which was estimated as $2(B_{\mathrm{rel}}^{1} - B_{\mathrm{rel}}^{0})$, as detailed in the methods section. However, this estimation yielded the possibility of misidentifying some non-single trajectories as single (false positives). In order to determine how many of these trajectories were false positives, we calculated a maximum false positive fraction (FPR) for each brightness distribution, where FPR = (number of trajectories misidentified as single) / (number of trajectories assigned as single). The number of misidentified trajectories was determined by assuming that the single-QD peak was symmetrical, which allowed us to estimate the expected number of single trajectories. This value could be subtracted from the total number of trajectories assigned as single to determine the number of trajectories misidentified as single. (a) Scatter plot of f_1 and FPR values. (b) Histograms of FPR values. Number of data points are indicated by the area under the histogram.

Supplementary Tables

Supplementary Table 1. Average diffusion coefficients and confinement parameters for data in Fig. 4a and 4e

	Single-Trajectory Analysis		Ensemble Analysis	
	$\bar{D}$ (μm^{2}s^{-1})	$\bar{\alpha}$	$\bar{D}$ (μm^{2}s^{-1})	$\bar{\alpha}$
mPEG	0.023	0.26	0.020	0.44
aCOOH	0.030	0.34	0.025	0.46
pCOOH	0.037	0.24	0.036	0.46
pPEG	0.090	0.37	0.083	0.54
pZW	0.22	0.49	0.23	0.70

Supplementary Table 2. FCS fit parameters for pPEG-QDs delivered to HeLa cells. Parameters correspond to those defined in Supplementary Equation 3.

	$\tau_{D,1}$(s)	D_1 (μm^2/s)	α_1	A_1	$\tau_{D,2}$(s)	D_2 (μm^2/s)	α_2	A_2	R^2
trace 1	0.0073	4.50	0.83	0.65	0.037	0.90	0.92	0.35	0.9957
trace 2	0.009	3.50	0.57	1.0	--	--	--	--	0.9979
trace 3	0.054	0.61	0.52	0.41	0.16	0.20	0.80	0.59	0.9818
trace 4	0.69	0.045	0.24	1.0	--	--	--	--	0.9900
trace 5	2.98	0.011	0.85	1.0	--	--	--	--	0.9893
trace 6	0.021	1.54	0.60	1.0	--	--	--	--	0.9984
trace 7	0.071	0.46	0.49	1.0	--	--	--	--	0.9980
trace 8	1.70	0.019	0.94	1.0	--	--	--	--	0.9936
trace 9	0.073	0.45	0.71	1.0	--	--	--	--	0.9798
trace 10	0.52	0.063	0.98	0.74	0.92	0.036	0.41	0.26	0.9901
trace 11	0.029	1.15	0.34	1.0	--	--	--	--	0.9976
trace 12	1.72	0.020	0.66	1.0	--	--	--	--	0.9825
trace 13	0.82	0.040	1.0	0.59	0.84	0.039	0.36	0.41	0.9681

Supplementary Table 3. *p*-values for f_{mobile}: QD coating comparison

	mPEG	aCOOH	pCOOH	pPEG	pZW
mPEG	1.00	0.92	0.44	0.0000014	0.00000000000047
aCOOH	0.92	1.00	0.42	0.0000031	0.0000000000017
pCOOH	0.44	0.42	1.00	0.0000053	0.00000000000013
pPEG	0.0000014	0.0000031	0.0000053	1.00	0.000012
pZW	0.00000000000047	0.0000000000017	0.00000000000013	0.000012	1.00

Supplementary Table 4. *p*-values for f_1: QD coating comparison

	mPEG	aCOOH	pCOOH	pPEG	pZW
mPEG	1.00	0.44	0.051	0.000050	0.00000082
aCOOH	0.44	1.00	0.25	0.000050	0.0000016
pCOOH	0.051	0.25	1.00	0.00016	0.0000031
pPEG	0.000050	0.000050	0.00016	1.00	0.59
pZW	0.00000082	0.0000016	0.0000031	0.59	1.00

Supplementary Table 5. *p*-values for $f_{1,mobile}$: QD coating comparison

	mPEG	aCOOH	pCOOH	pPEG	pZW
mPEG	1.00	0.097	0.056	0.00000068	0.00000000066
aCOOH	0.097	1.00	0.53	0.000019	0.000000016
pCOOH	0.056	0.53	1.00	0.0000052	0.000000000058
pPEG	0.00000068	0.000019	0.0000052	1.00	0.0048
pZW	0.00000000066	0.000000016	0.000000000058	0.0048	1.00

Supplementary Table 6. *p*-values for N_{cell}: QD coating comparison

	mPEG	aCOOH	pCOOH	pPEG	pZW
mPEG	1.00	0.0062	0.021	0.0000012	0.00000000011
aCOOH	0.0062	1.00	0.0072	0.49	0.086
pCOOH	0.021	0.0072	1.00	0.00000014	0.0000000000015
pPEG	0.0000012	0.49	0.00000014	1.00	0.00026
pZW	0.00000000011	0.086	0.0000000000015	0.00026	1.00

Supplementary Table 7. *p*-values for N_{mobile}: QD coating comparison

	mPEG	aCOOH	pCOOH	pPEG	pZW
mPEG	1.00	0.066	0.15	0.084	0.42
aCOOH	0.066	1.00	0.020	0.00035	0.0031
pCOOH	0.15	0.020	1.00	0.51	0.086
pPEG	0.084	0.00035	0.51	1.00	0.14
pZW	0.42	0.0031	0.086	0.14	1.00

Supplementary Table 8. *p*-values for f_{mobile}: cell type comparison

	CHO	HeLa	single A431	clumped A431
CHO	1.00	0.38	0.43	0.21
HeLa	0.38	1.00	0.045	0.55
single A431	0.43	0.045	1.00	0.066
clumped A431	0.21	0.55	0.066	1.00

Supplementary Table 9. *p*-values for f_1: cell type comparison

	CHO	HeLa	single A431	clumped A431
CHO	1.00	0.00010	0.014	0.089
HeLa	0.00046	1.00	0.19	0.037
single A431	0.014	0.19	1.00	0.47
clumped A431	0.089	0.037	0.47	1.00

Supplementary Table 10. *p*-values for $f_{1,mobile}$: cell type comparison

	CHO	HeLa	single A431	clumped A431
CHO	1.00	0.0038	0.028	0.57
HeLa	0.0038	1.00	0.52	0.052
single A431	0.028	0.52	1.00	0.18
clumped A431	0.57	0.052	0.18	1.00

Supplementary Table 11. *p*-values for N_{cell}: cell type comparison

	CHO	HeLa	single A431	clumped A431
CHO	1.00	0.000010	0.17	0.69
HeLa	0.000010	1.00	0.00082	0.00049
single A431	0.17	0.00082	1.00	0.73
clumped A431	0.69	0.00049	0.73	1.00

Supplementary Table 12. *p*-values for N_{mobile}: cell type comparison

	CHO	HeLa	single A431	clumped A431
CHO	1.00	0.031	0.32	0.63
HeLa	0.031	1.00	0.015	0.069
single A431	0.32	0.015	1.00	0.86
clumped A431	0.63	0.069	0.86	1.00

Supplementary Methods

Optical Spectroscopy. Fluorescent spectra were measured using a NanoLog Horiba Jobin Yvon with Fluo Essence V3.5 software (HORIBA Scientific). UV-Vis spectra were obtained using a Cary series UV-Vis-NIR spectrophotometer with Cary WinUV Scan Application Version 6.00 1551 software (Agilent Technologies).

Transmission Electron Microscopy of QDs. TEM images were obtained using a JEOL 2010 LaB6 high-resolution microscope in the Frederick Seitz Materials Research Laboratory Central Research Facilities at University of Illinois. For QDs in organic solvents, samples were prepared by placing a drop of dilute QD solution in hexane on an ultrathin carbon film TEM grid (Ted Pella; Product # 01824) and then wicking the solution off with a tissue.

DLS and Zeta Potential. Dynamic light scattering and zeta potentiometry were performed using a Zetasizer Nano ZS (Malvern Instruments Ltd.) with samples dispersed in 10 mM phosphate buffer (pH 7.4).

Transmission Electron Microscopy of Cells. Cells were cultured as monolayers on multi-well plates and fixed overnight at 4°C with 2.5% glutaraldehyde in 0.1 M cacodylate buffer (pH 7.4). Cells were then washed with the same buffer and post- fixed in 1% osmium tetroxide with 1.5% potassium ferrocyanide in the same buffer for one hour. The samples were subsequently rinsed with 2 or 3 exchanges of de-ionized water, dehydrated through an ethanol series ending with three exchanges of 100% absolute ethanol, and then embedded in Eponate 12 resin (Ted Pella, Inc.) by placing resin-infiltrated cells in a 60°C oven for 2 days. Upon resin polymerization, hardened resin blocks with monolayer cells on the bottom surface were removed from the culture plate, sawed into smaller pieces, and thin-sectioned parallel to the cell surface at 70 nm. Sections were then picked up with 200 mesh copper grids, stained with 5% aqueous uranyl acetate and 2% lead citrate, and viewed on a Hitachi H-7500 transmission electron microscope (Hitachi High Technologies America, Inc.) equipped with a BioScan CCD camera (Gatan, Inc.).

pPEG and pCOOH Quantum Dot Coating. A methanol solution of tetramethylammonium hydroxide (25%) was added to a biphasic mixture of *N*-methylformamide (NMF) and purified QDs in hexane. The suspension was stirred vigorously for 1 hour until the QDs were completely transferred to the NMF phase. Hexane was removed, and the NMF solution was washed with hexane twice. Residual hexane and methanol were evaporated under vacuum. A solution of pPEG or pCOOH in NMF (5:1 imidazole to QD surface atom) was added dropwise to the hydroxide-coated QDs in NMF (1 µM, 0.4 mL) with stirring under nitrogen atmosphere. The reaction was allowed to proceed at 110°C for 2 hours. The coated QDs were precipitated from NMF using anhydrous diethyl ether. The QDs were then dispersed in 50 mM sodium borate buffer (pH 8.5) and centrifuged to remove possible aggregates. The QDs were purified using centrifugal filtration (Amicon Ultra 50 kDa molecular weight cutoff) in 50 mM sodium borate buffer. The dilution–filtration cycle was performed five times.

pZW Quantum Dot Coating. Purified QDs in hexane were transferred to NMF using the same method as for the pPEG and pCOOH polymers. pZW polymer dissolved in NMF was mixed with the hydroxide-coated QDs in NMF (1 µM, 0.4 mL) (5:1 thiol to QD surface atom) and purged with nitrogen for 2 minutes. The mixture was stirred at 110°C for 4 hours and then diluted with 50 mM sodium borate buffer (pH 8.5). The QDs were purified using centrifugal filtration (50 kDa molecular weight cutoff) in 50 mM sodium borate buffer. The dilution–filtration cycle was performed five times.

aCOOH Quantum Dot Coating*.* Purified QDs were dispersed in chloroform (~1 µM, 2-10 mL) and mixed with a 2,000-2,500-fold molar excess of aCOOH. Chloroform was slowly evaporated under vacuum with vigorous stirring. After complete evaporation, a 10 mM sodium hydroxide solution in distilled water (2–3 mL $nmol^{-1}$) was added and stirred for several hours until the amphipol-coated QDs were fully dispersed. Finally, the solution was centrifuged to remove possible aggregates.

mPEG-QD Stability. The stability of mPEG coated QDs in 50 mM sodium borate buffer was measured by centrifugation at 7000*g* for 10 minutes to remove any aggregates that may have formed due to coating instability. Concentrations were determined by measuring absorbance at 350 nm using a NanoDrop ND-1000 Spectrophotometer (Thermo Fisher Scientific).

Bromocresol Green (BCG) Quenching. Quenching of QDs by BCG was measured using a Synergy HT microplate reader (BioTek Instruments, Inc.). Aqueous QDs (10 nM in 50 mM borate buffer, pH 8.5) were mixed in triplicate with 0, 1, 4.16, 17.3, 72.1, and 300 µM BCG (Sigma Aldrich). The ratio of fluorescence intensity at each BCG concentration to the zero-BCG sample (I/I_o) was calculated by measuring the fluorescence of each sample at 400 nm excitation and 600 nm emission.

Localization Error. QDs were immobilized on a #1.5 coverslip and imaged identically to those in cells using HILO, as described in the *Methods* section. After SPT analysis of these images, diffusion coefficients were derived from *MSD* curves fit to an anomalous diffusion model for the first 10 time-increments to determine the localization error of our instrumentation and any error that may derive from an artifact of the analysis technique, which was determined to be 0.02 $\mu m^2/s$.

SPT in Solution. For SPT in cell-free solutions, QDs in 50 mM sodium borate buffer were diluted to 0.123 nM with glycerol to reach a final glycerol concentration of 98%. Approximately 100 µL of this QD dispersion was imaged on a #1.5 coverslip. HILO images of freely diffusing QDs were acquired as described above (see *Localization Error*). After SPT analysis of these images, diffusion coefficients were derived from *MSD* curves fit to an anomalous diffusion model for the first 10 time-increments. For accurate calculation of hydrodynamic diameter, we only used particle tracks with a minimum length of 200 frames and a diffusion coefficient greater than 0.02 $\mu m^2/s$ – the aforementioned empirically determined localization error for our imaging system.[2,3] Hydrodynamic diameters were calculated using the Stokes-Einstein equation and known viscosity values of glycerol solutions.[4]

Lipofectamine Delivery of Quantum Dots. HeLa cells (ATCC) were seeded at a density of 25,000 cells cm^{-2} in LabTek chambers (Thermo Scientific), 24 hours before delivery. For each well, Lipofectamine 2000 (4 µL; Life Technologies) was incubated with pPEG-coated QD605 (10 µL; ~6 nM) in 100 µL incomplete DMEM without phenol red for 20 minutes to prepared QD-Lipofectamine complexes.[5] Then 100 µL of the mixture was added to each well and incubated with the cells for ~3 hours. The medium was replaced with complete DMEM without phenol red and the cells were incubated for 30 minutes at 37°C. Nuclei were stained with Hoechst (Sigma-Aldrich) for 20 minutes, followed by washing and treatment with BCG (200 µM) in phenol red-free DMEM to quench any extracellular QDs. Cells were imaged within 4 hours after initial addition of QDs to cells.

Cytotoxicity Studies. CHO cells were seeded in a 96-well plate (Greiner Bio-One) at a density of 20,000 cells per well, 24 hours prior to treatments. For the cytotoxicity study, 10 µL of Cell Counting Kit-8 (Dojindo Molecular Techniques, Inc.) was added to each well. After 3 hours, the absorbance of each well was measured at 450 nm using a Synergy HT microplate reader (BioTek Instruments, Inc.). For the viability study, 2 µM calcein AM (Santa Crux Biotechnology, Inc.) and 4 µM ethidium homodimer-1 (Setareh Biotech) were added to each well and incubated for 15 minutes at 37°C. Live/dead imaging was performed with a 20× 0.50 NA Plan-Neoufluar dry objective. Both calcein AM and ethidium homodimer-1 were excited using a 100 W halogen lamp, with excitation and emission light filtered by GFP and Cy3 filter sets (Zeiss), respectively. For the DAPI membrane-permeability measurement, CHO cells seeded at a density of 40,000 cells mL^{-1} in a LabTek chamber were loaded with 40 nM pPEG QDs with standard parameters of 10 minute hypertonic loading and 3 minute hypotonic lysis. The cells were then incubated with 1 µg/mL of DAPI (Sigma Aldrich), which allowed us to distinguish live cells from dead cells. Then we located cells with and without DAPI stain and acquired HILO images of the intracellular QDs and performed standard SPT and diffusion analysis.

Nonspecific Adsorption Experiments. HeLa cells were seeded at a density of 72,000 cells/cm^2 in CellView dishes (VWR), 24 hours before OPL treatment. Cells were washed twice with phosphate buffered saline, and the hypertonic loading reagent (Life Technologies) containing mPEG-QDs (10 nM) with or without 0.5× casein blocking buffer (Sigma-Aldrich) was added. Cells were incubated for 10 minutes at 37°C, and the medium was removed and replaced with hypotonic lysis medium. All subsequent steps for OPL delivery described in the main text methods were then followed.

FCS Studies. HeLa cells were seeded at a density of ~84,000 cells/cm^2 in glass-bottom CellView dishes (Greiner Bio-One) 24 hours before OPL delivery of 240 nM pPEG-QDs. FCS data were acquired on an Alba FCS instrument (ISS) with a diode laser (470 nm) for excitation and single-photon avalanche photodiode detector. Each trace was acquired for 30 seconds at a frequency of 100,000 Hz.

To measure the confocal spot size of the FCS instrument, FCS data were acquired for a dye standard with known diffusion coefficient (Rhodamine 110; $D = 17.6\times10^{-10}$ m^2s^{-1}), and the resulting autocorrelation function, $G(\tau)$, was fit to the following equation:

$$G(\tau) = \frac{1}{N}\frac{\left[1 - F + F\exp\left(-\frac{\tau}{\tau_{triplet}}\right)\right]}{1 - F}\left(1 + \frac{\tau}{\tau_D}\right)^{-1}\left[1 + \frac{\tau}{\tau_D}\left(\frac{\omega_{xy}}{\omega_z}\right)^2\right]^{-\frac{1}{2}} \qquad (1)$$

where N is the average number of particles in the confocal volume, F is the triplet fraction, $\tau_{triplet}$ is the characteristic blinking time of the dye, τ_D is the characteristic decay time associated with the diffusion of the dye through the confocal volume, ω_{xy} is the xy-radius of the confocal spot, and ω_z is the z-radius of the confocal spot.[6] The spot sizes of ω_{xy} = 0.363 μm and ω_z = 3.07 μm were used as fixed parameters for subsequent FCS curve fittings.

To measure the characteristic blinking time for our QDs (τ_{blink}), FCS data for pPEG-coated QDs in aqueous buffer were acquired, and the resulting autocorrelation function was fit to the following equation:

$$G(\tau) = \frac{1}{N}\left[1 + \theta\exp\left(-\frac{\tau}{\tau_{triplet}}\right)\right]\left(1 + \frac{\tau}{\tau_D}\right)^{-1}\left[1 + \frac{\tau}{\tau_D}\left(\frac{\omega_{xy}}{\omega_z}\right)^2\right]^{-\frac{1}{2}} \qquad (2)$$

where θ is a factor that accounts for the number of particles in a nonfluorescent state.[7] The result of this measurement was τ_{blink}= 187 μs, which was used as a fixed parameter for subsequent fitting of FCS curve fittings.

Autocorrelation functions for pPEG-QDs in cells were fit to a multicomponent anomalous diffusion model shown below:

$$G(\tau) = \frac{1}{N}\left[1 + \theta\exp\left(-\frac{\tau}{\tau_{triplet}}\right)\right]\sum_n A_n\left[1 + \left(\frac{\tau}{\tau_{D,n}}\right)^{\alpha_n}\right]^{-1}\left[1 + \left(\frac{\tau}{\tau_{D,n}}\right)^{\alpha_n}\left(\frac{\omega_{xy}}{\omega_z}\right)^2\right]^{-\frac{1}{2}} \qquad (3)$$

where α_n is the confinement parameter for the nth component, and A_n is the fractional contribution the nth component to the total curve. Each fit was calculated with 1 to 4 components. Minima of the Akaike information criteria (AIC) were used to determine the number of fitted components.

The diffusion coefficient D_n for each component was calculated by using the following equation:[8]

$$D_n = \frac{\omega_{xy}^2}{4\tau_{D,n}} \qquad (4)$$

Statistical Analysis. All error bars are standard error of the mean values, unless indicated otherwise. All *p*-values were calculated using a two-tailed *t*-test, unless indicated otherwise.

Supplementary References

1 Yi, H., Leunissen, J., Shi, G., Gutekunst, C. & Hersch, S. A novel procedure for pre-embedding double immunogold-silver labeling at the ultrastructural level. *J. Histochem. Cytochem.* **49**, 279-284 (2001).

2 Saxton, M. J. & Jacobson, K. Single-particle tracking: applications to membrane dynamics. *Annu. Rev. Biophys. Biomol. Struct.* **26**, 373-399 (1997).

3 Ernst, D. & Kohler, J. Measuring a diffusion coefficient by single-particle tracking: statistical analysis of experimental mean squared displacement curves. *Phys. Chem. Chem. Phys.* **15**, 845-849 (2013).

4 Segur, J. B. & Oberstar, H. E. Viscosity of Glycerol and Its Aqueous Solution. *Ind. Eng. Chem.* **43**, 5-8 (1951).

5 Voura, E. B., Jaiswal, J. K., Mattoussi, H. & Simon, S. M. Tracking metastatic tumor cell extravasation with quantum dot nanocrystals and fluorescence emission-scanning microscopy. *Nat. Med.* **10**, 993-998 (2004).

6 Bachir, A. I., Kolin, D. L., Heinze, K. G., Hebert, B. & Wiseman, P. W. A guide to accurate measurement of diffusion using fluorescence correlation techniques with blinking quantum dot nanoparticle labels. *J. Chem. Phys.* **128**, 225105 (2008).

7 Bestvater, F. *et al.* EMCCD-based spectrally resolved fluorescence correlation spectroscopy. *Opt. Express* **18**, 23818-23828 (2010).

8 Banks, D. S., Tressler, C., Peters, R. D., Höfling, F. & Fradin, C. Characterizing anomalous diffusion in crowded polymer solutions and gels over five decades in time with variable-lengthscale fluorescence correlation spectroscopy. *Soft Matter* **12**, 4190-4203 (2016).
