# Supplementary material for: Single quantum dot tracking reveals the impact of nanoparticle surface on intracellular state
Source: Nat Commun. 2018 May 8;9:1830. doi: 10.1038/s41467-018-04185-w (PMC5940692; doi:10.1038/s41467-018-04185-w)
Supplement: Supplementary file 2 — Description of Additional Supplementary Files [file 41467_2018_4185_MOESM2_ESM.pdf]

Description of Additional Supplementary Files

File Name: Supplementary Movie 1
Description: Chinese hamster ovary cell after mPEG quantum dot delivery. Representative still image shows brightfield micrograph with nuclear stain overlay (blue). The corresponding real-time HILO movie shows mPEG-coated quantum dots delivered by osmotic pinosome lysis. High levels of clustering and a large immobile fraction are apparent.

File Name: Supplementary Movie 2
Description: Chinese hamster ovary cell after aCOOH quantum dot delivery. Representative still image shows brightfield micrograph with nuclear stain overlay (blue). The corresponding real-time HILO movie shows aCOOH-coated quantum dots delivered by osmotic pinosome lysis. High levels of clustering and a large immobile fraction are apparent.

File Name: Supplementary Movie 3
Description: Chinese hamster ovary cell after pCOOH quantum dot delivery. Representative still image shows brightfield micrograph with nuclear stain overlay (blue). The corresponding real-time HILO movie shows pCOOH-coated quantum dots delivered by osmotic pinosome lysis. Lower levels of clustering with a large immobile fraction are apparent.

File Name: Supplementary Movie 4
Description: Chinese hamster ovary cell after pPEG quantum dot delivery. Representative still image shows brightfield micrograph with nuclear stain overlay (blue). The corresponding real-time HILO movie shows pPEG-coated quantum dots delivered by osmotic pinosome lysis. Low levels of clustering and a high mobile fraction are apparent.

File Name: Supplementary Movie 5
Description: Chinese hamster ovary cell after pZW quantum dot delivery. Representative still image shows brightfield micrograph with nuclear stain overlay (blue). The corresponding real-time HILO movie shows pZW-coated quantum dots delivered by osmotic pinosome lysis. Low levels of clustering and a large mobile fraction are apparent.

File Name: Supplementary Movie 6
Description: HeLa cell after mPEG quantum dot delivery. Representative still image shows brightfield micrograph with nuclear stain overlay (blue). The corresponding real-time HILO movie shows mPEG-coated quantum dots delivered by osmotic pinosome lysis without blocking. Higher levels of QD clustering and a small fraction of mobile quantum dots are apparent compared with those delivered with blocking in Supplementary Movie 7.

File Name: Supplementary Movie 7
Description: HeLa cell after mPEG quantum dot delivery. Representative still image shows brightfield micrograph with nuclear stain overlay (blue). The corresponding real-time HILO movie shows mPEG-coated quantum dots delivered by osmotic pinosome lysis with blocking. Lower levels of QD clustering and a high fraction of mobile quantum dots are apparent compared with those delivered without blocking in Supplementary Movie 6.
